# Anterior–posterior and medial-lateral balance metrics are unchanged when two-dimensional pseudorandom motion perturbations are provided in semicircular canal coordinates

**DOI:** 10.3389/fneur.2025.1638493

**Published:** 2025-08-25

**Authors:** Manami Fujii, Sophia G. Chirumbole, Andrew R. Wagner, Jaclyn B. Caccese, Ajit M. W. Chaudhari, Wei Wang, Bo Lu, Daniel M. Merfeld

**Affiliations:** ^1^Otolaryngology—Head and Neck Surgery, The Ohio State University Wexner Medical Center, Columbus, OH, United States; ^2^Biomedical Engineering, The Ohio State University, Columbus, OH, United States; ^3^Mechanical and Aerospace Engineering, The Ohio State University, Columbus, OH, United States; ^4^Department of Physical Therapy, Creighton University, Omaha, NE, United States; ^5^School of Health and Rehabilitation Sciences, The Ohio State University, Columbus, OH, United States; ^6^Departments of Medicine and Neurology, Division of Sleep and Circadian Disorders, Brigham and Women's Hospital, Boston, MA, United States; ^7^Division of Sleep Medicine, Harvard Medical School, Boston, MA, United States; ^8^Division of Biostatistics, The Ohio State University, Columbus, OH, United States; ^9^Speech and Hearing Science, The Ohio State University, Columbus, OH, United States

**Keywords:** postural control, balance, posturography, vestibular, pseudorandom perturbations, sum of sines, semicircular canals, motion platform

## Abstract

**Introduction:**

External continuous perturbations using a motion platform have been developed by employing either sum-of-sines (SoS) or a pseudorandom ternary sequence (PRTS) of numbers to quantify body sway evoked in the medial-lateral (ML) or anterior-posterior (AP) directions, which ultimately helps understand the human postural control system. These stimuli have been provided via pitch tilts of the motion platform for evaluations of AP balance responses or roll tilts for ML balance responses. However, little is known about whether a healthy postural control system responds to 2-dimensional (2D) perturbations similarly when the perturbation stimuli are provided in semicircular canal coordinates (i.e., right-anterior/left-posterior (RALP) and left-anterior/right-posterior (LARP)) versus roll/pitch coordinates. Stimuli provided in either set of coordinates were orthogonal in both time and space. Our 2D platform perturbations provided in RALP/LARP coordinates will have the potential to better assess the contribution of each pair of the vertical semicircular canals to postural control for individuals with dysfunction of the vertical semicircular canals.

**Methods:**

To address this knowledge gap, we developed four different balance perturbation trajectories using sum-of-sines (SoS) signals and simultaneously provided those stimuli in (i) roll and pitch, (ii) RALP and LARP, and (iii) roll, pitch, RALP, and LARP dimensions. Center of pressure (CoP) data were collected from 24 healthy participants (40 13 years of age) on a commercially available motion platform (Virtualis Motion VR, Perault, France). A discrete Fourier transform (DFT) was applied to the CoP data to identify responses at perturbed frequencies (i.e., spectral response components).

**Results:**

We found that ML and AP postural responses were not significantly different when the platform perturbations were simultaneously provided in RALP/LARP coordinates versus roll/pitch coordinates.

**Discussion:**

This finding suggests that our 2D platform perturbations in RALP/LARP coordinates allow us (1) to compare ML and AP responses evoked by RALP and LARP stimuli to existing literature showing those responses evoked by roll and pitch stimuli and (2) to characterize postural responses for individuals with sensory deficits to better isolate contributions of the vertical semicircular canals to postural control.

## Introduction

Dating back to the original Neurocom platforms (1–3), sway-referenced tilts of a rigid support surface are a fundamental element of the sensory organization test (SOT), which remains a standard clinical balance test (4, 5) that is often used for research (2, 6, 7). More recently, steady-state sway responses to continuous perturbations introduced using either sums-of-sines (SoS) stimuli (8–13) or pseudorandom ternary sequence (PRTS) (14–18) stimuli have been quantified to provide spectral information about reactive postural control (14, 17, 19–24). A standard perturbation approach is to apply 1-dimensional (1D) pitch stimuli to quantify anterior–posterior (AP) sway response (25–31). Less commonly, 1D roll stimuli are also provided to quantify medial-lateral (ML) sway response (12, 20, 32, 33).

Other studies suggest that AP and ML balance response templates and muscle synergies (34, 35) exist as part of the nervous system’s postural control system (36). By this logic, the nervous system appears to maintain separate control of AP balance responses and ML balance responses, which are orthogonal to one another in space. The body sways in ML differently than in AP to maintain balance, which is related to an engineering concept called controllability (37).

We have noted that 1D motion stimuli cannot characterize the influence of vestibular impairment on postural control in the remaining unperturbed directions (38). While less common than 1D stimuli, multiple simultaneous stimuli (i.e., multidimensional stimuli) have been used to study reactive balance control (25, 31, 39–43), and we recently introduced and tested a multidimensional SOT balance test (7) as well as a multidimensional pseudorandom balance perturbation test that provided simultaneous roll and pitch perturbations of the rigid support surface on which the participant stood (38). In our 2D perturbation study, we tilted the support surface separately using pseudorandom SoS stimuli in roll or pseudorandom SoS stimuli in pitch and compared the AP and ML response components to those evoked by simultaneous 2D SoS stimuli designed to have separable spectra for roll and pitch. We reported that the responses evoked by the 2D tilt of the support surface (i.e., roll and pitch together) were generally not different from those evoked by 1D tilts of the support surface (roll or pitch on separate trials).

While this study successfully applied spectrally isolated and spatially orthogonal tilt stimuli in both roll and pitch directions, these multidimensional balance perturbation methods suggest new questions—e.g., “In what directions should we apply motion stimuli?” and “Might motion stimuli in planes other than roll and pitch be beneficial?” This study begins to address such questions.

**Figure 1:**
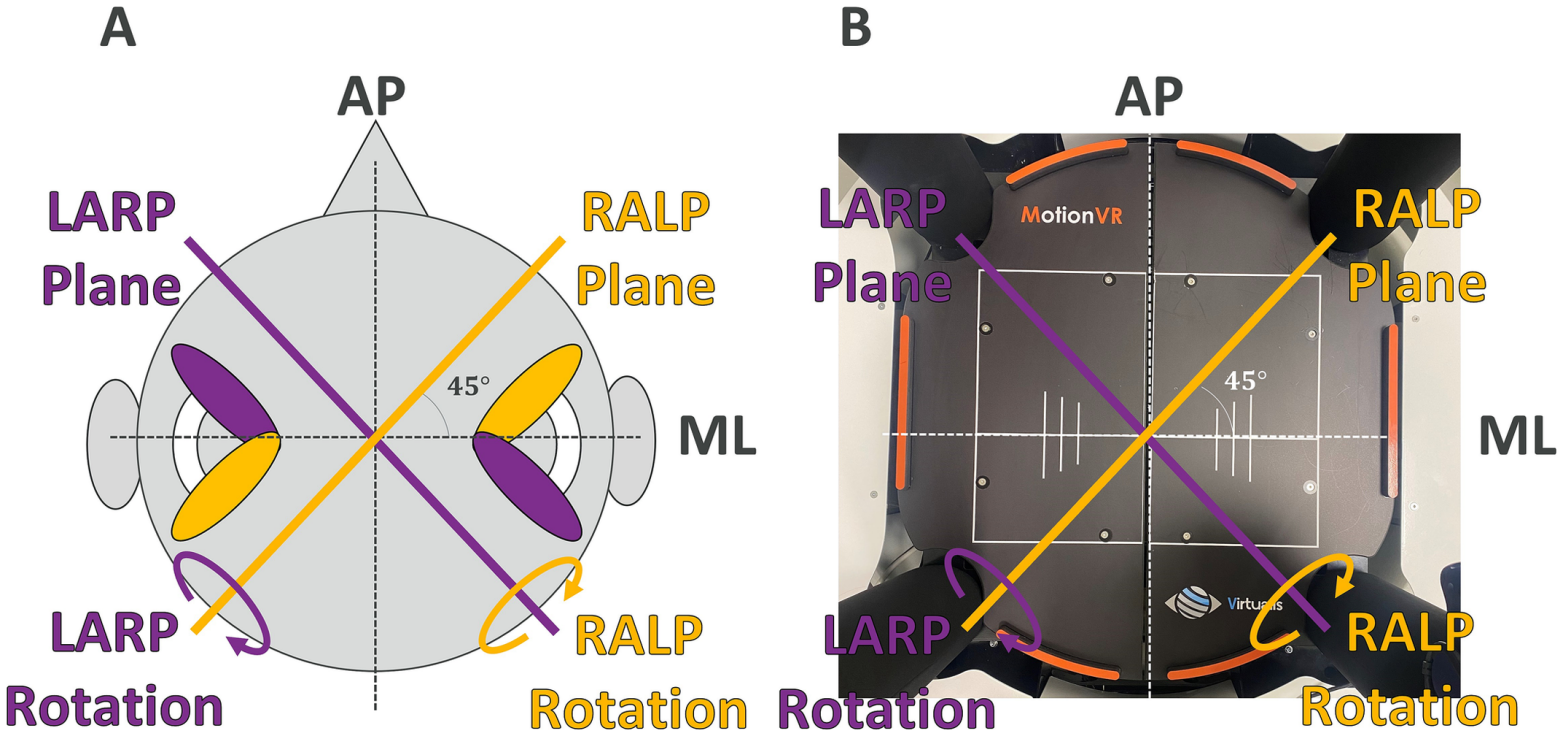
RALP (right anterior and left posterior) and LARP (left anterior and right posterior) planes of the head **(A)** and the Virtualis platform **(B)** are shown. The rotation that maximally stimulates the RALP canals (and minimally stimulates the LARP canals) is in the LARP plane as shown. The rotation that maximally stimulates the LARP canals (and minimally stimulates the RALP canals) is in the RALP plane as shown.

While balance testing can (and often does) consider the sensory contributions of vestibular function to balance (6, 17, 23), vestibular testing often focuses exclusively on the contributions (i.e., transduction and sensory processing) of vestibular pathways to measured behaviors such as the vestibulo-ocular reflexes (VOR). For example, a common vestibular test is a head-impulse test (HIT) that assays the VOR evoked by head angular velocity impulses (44). These impulses are provided about one of three roughly orthogonal axes that provide rotations in the planes of the semicircular canals, including (i) yaw head rotations in the plane of the lateral semicircular canals, or rotations in the plane of (ii) the right-anterior/left-posterior (RALP) semicircular canal pair or (iii) the left-anterior/right-posterior (LARP) semicircular canal pair (Figure 1). Providing these head rotations in the planes of the semicircular canals focuses on the contributions of each of these three functional semicircular canal pairs to the VOR.

As one specific clinical example, consider an individual who has lost information from the left posterior semicircular canal (i.e., functional lesions of the left-posterior canal) but has fully functioning right anterior semicircular canal, LARP semicircular canals, and lateral semicircular canals. Robust responses from the LARP canals would result when roll rotations or pitch rotations are provided because the LARP canals respond to both roll and pitch stimuli, which have substantive projections onto the functioning LARP canals. On the other hand, the LARP semicircular canals would minimally respond to rotations in the orthogonal RALP canal plane (45), so a left posterior semicircular canal deficit is easy to observe in the VOR response when stimuli are provided in RALP/LARP coordinates but not as easy to observe when stimuli are provided in roll/pitch coordinates. More specifically, the left posterior semicircular canal deficit might lead to increasing postural sway in the RALP plane when the stimuli are provided in RALP/LARP coordinates while leading to increasing postural sway in both the roll and pitch planes when the stimuli are provided in roll/pitch coordinates. Providing stimuli in the canal coordinates makes sensory deficits easier to observe, which relates to an engineering concept called observability (37).

The idea of assessing dynamic behavior in response to RALP/LARP platform tilts leads to fundamental questions. For example, “When performing multidimensional balance perturbation testing, should we provide 2D tilt stimuli in roll/pitch coordinates or RALP/LARP coordinates?” Providing 2D stimuli in RALP/LARP coordinates has the benefit of making it easier to observe sensory deficits that arise from the vertical semicircular canals. But, if we provide tilt stimuli in the RALP/LARP coordinates, can we quantify the AP responses and ML responses evoked by these RALP/LARP stimuli and get results that are not different from those obtained if the stimuli had been provided in roll and pitch? More specifically, are the AP and ML responses evoked by RALP and LARP stimuli significantly different from AP and ML responses quantified using 2D stimuli provided as roll and pitch for healthy adults? If not significantly different, this might allow the application of 2D pseudorandom perturbations in RALP/LARP coordinates—making sensory impacts easier to measure—while still maintaining assessment of AP and ML response components, which would help maintain the ability to quantify impacts on known AP and ML motor templates (34, 35).

To address these fundamental questions, we compared AP and ML response components when 2D motion stimuli were simultaneously provided in roll/pitch coordinates to the AP and ML response components evoked when 2D stimuli were simultaneously provided in RALP/LARP coordinates. Our hypotheses were: (1) that AP and ML responses can be quantified when the 2D stimuli are provided in RALP/LARP coordinates and (2) that the AP and ML responses are significantly different when the stimuli are provided in roll/pitch coordinates versus RALP/LARP coordinates.

These are the fundamental hypotheses studied herein, but, in addition, we use these data to address a secondary question. Earlier findings have clearly established that responses to balance perturbations vary systematically with frequency (15). To help establish the sensitivity of the multidimensional perturbation methods to small frequency changes, we evaluated the impacts of small frequency changes on the postural responses utilizing 4 different SoS signals that include small differences in the frequency at which each spectral component appears. The data rejected our hypothesis that small frequency changes would not matter because the response variability would be greater than the small incremental systematic impacts of small frequency effects, suggesting that response variability does not mask these subtle influences.

Finally, we also added a test condition that applied four SoS signals in the roll, pitch, RALP, and LARP directions simultaneously. We hypothesized that postural responses would include postural responses to each of the four simultaneous SoS perturbations—each providing platform stimuli at five different frequencies, which proved correct.

In summary, the research questions addressed in this study are (1) whether AP and ML responses can be quantified when the 2D stimuli are provided in RALP/LARP coordinates, (2) whether the AP and ML responses are significantly different when the stimuli are provided in roll/pitch coordinates versus RALP/LARP coordinates, (3) whether small changes in perturbation frequency matter for postural responses, and (4) whether spectral postural response components are observed at each of 20 total different perturbation frequencies when four SoS perturbations are provided in roll, pitch, RALP, and LARP directions simultaneously. Our hypotheses are (1) AP and ML responses can be quantified even when the 2D stimuli are provided in RALP/LARP coordinates, (2) the AP and ML responses are not statistically different regardless if the stimuli are provided in roll/pitch coordinates or RALP/LARP coordinates, (3) small changes in perturbation frequency does not matter for postural responses, (4) spectral postural response components can be observed at all perturbed frequencies when the stimuli are provided in roll, pitch, RALP, and LARP directions simultaneously. The study aims to solidify our anatomically driven approach (i.e., RALP and LARP) to balance assessments in healthy controls before proceeding to patient assays.

## Methods

### Generation of the sum-of-sines (SoS) signals

SoS signals were delivered to perturb balance simultaneously in the roll and pitch directions during Condition (i), simultaneously in the RALP and LARP directions during Condition (ii), and in the roll, pitch, RALP and LARP directions simultaneously during Condition (iii). The roll and pitch signals were orthogonal to one another in both time and space. Similarly, the RALP and LARP signals were also orthogonal to one another in time and space. During Condition (iii), the roll, pitch, RALP and LARP stimuli were each orthogonal in time to all other stimuli, but roll and pitch stimuli were, by definition, not spatially orthogonal to the RALP and LARP stimuli.

Building on our earlier work that included two simultaneous SoS perturbations (38), we developed four distinct SoS balance perturbation trajectories that could be provided simultaneously during Condition (iii) as the stimuli for roll, pitch, RALP, and LARP. The trajectories used a fundamental frequency of 0.0055 Hz multiplied by four groups of interleaved multipliers ([11,59,109,179,241], [13,61,113,181,251], [15,71,131,193,263], and [17,67,127,191,257]). No multipliers were repeated; each multiplier, except 15, was a prime number. Neither of 15’s prime factors (3 or 5) was used; this avoids overlapping at higher harmonics, (e.g., 3rd harmonic associated with the multiplier 5). Each frequency component was orthogonal in time to all other frequency components. Each of these SoS trajectories was named to reflect the lowest multiplier (i.e., SoS_11_, SoS_13_, SoS_15_, and SoS_17_). When multiplied by the fundamental frequency, these integer sets yielded the following four frequency sets:


$$f_{\mathrm{SoS}11}=[0.0604,0.3241,0.5988,0.9833,1.3239]\;\mathrm{Hz}$$

$$f_{\mathrm{SoS}13}=[0.0714,0.3351,0.6207,0.9943,1.3788]\;\mathrm{Hz}$$

$$f_{\mathrm{SoS}15}=[0.0824,0.3900,0.7196,1.0602,1.4447]\;\mathrm{Hz}$$

$$f_{\mathrm{SoS}17}=[0.0934,0.3680,0.6976,1.0492,1.4117]\;\mathrm{Hz}$$

The four steady-state SoS displacement trajectories were generated by summing sinusoidal signals. For example, the following equation was used to generate the SoS_11_ trajectory:


$$\mathrm{So}S_{11}(t)=\sum_{i=1}^{5}A_{11,i}\sin(2\pi f_{\mathrm{SoS}11,i}t+\emptyset_{\mathrm{SoS}11,i})$$

In this equation, t represents time, $A_{11,i}\;$ is the ith amplitude, $f_{11,i}\;$ is the ith frequency, and $\emptyset_{11,i}\;$ is the ith phase value. These equations resulted in four SoS trajectories having interleaved, spectrally separated perturbation frequencies. Supplementary material shows the other three equations used to generate the rest of the trajectories (i.e., SoS_13_, SoS_15_, and SoS_17_).

**Figure 2:**
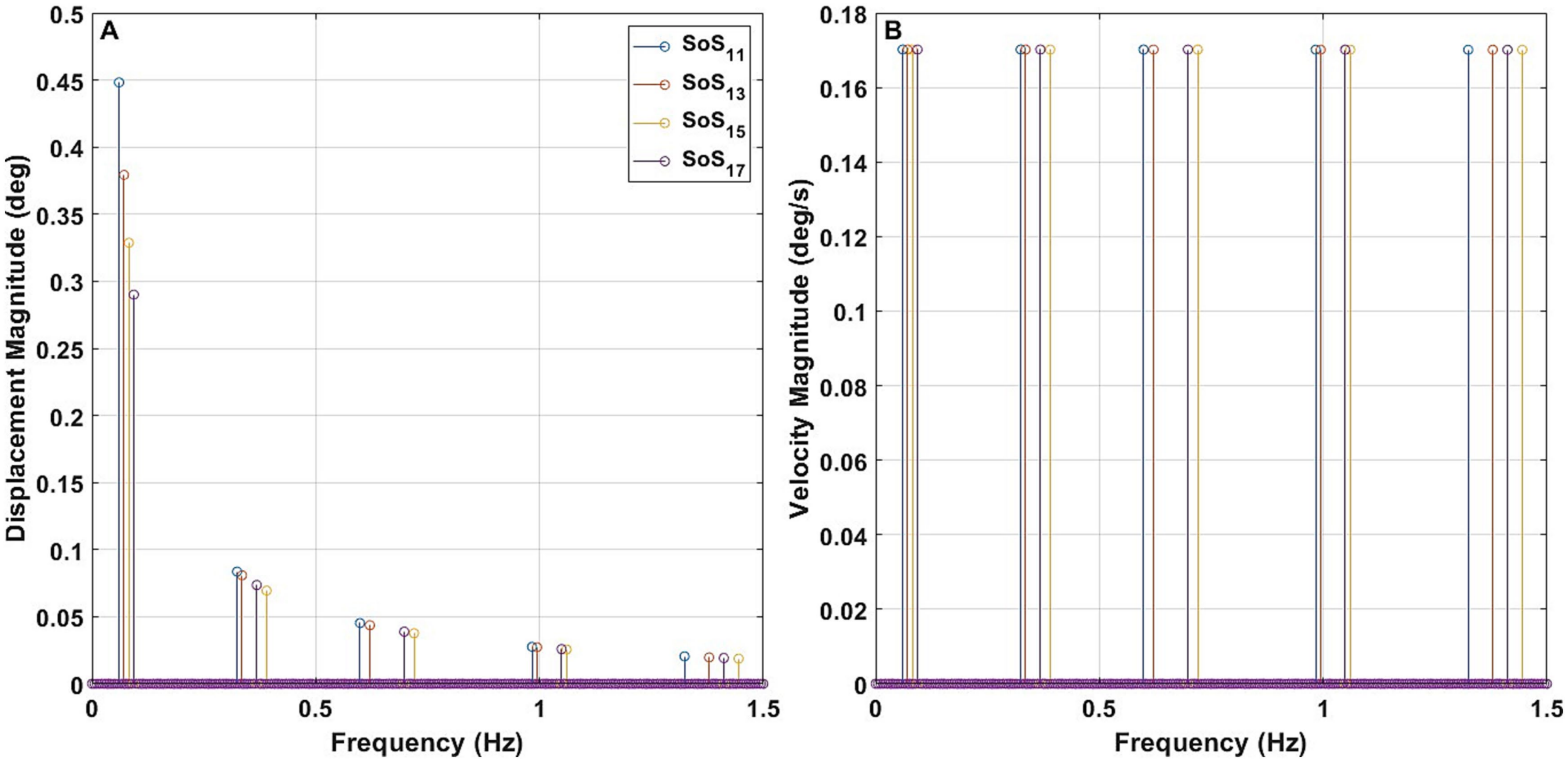
The displacement magnitude **(A)** and velocity magnitude **(B)** of the sum of sines (SoS_11_, SoS_13_, SoS_15_, and SoS_17_) trajectories at individual perturbation frequencies.

The phase values were determined via a systematic search using one-degree phase increments between 0 and 359° to minimize differences in the peak-to-peak amplitude of the four trajectories while keeping the velocity spectral magnitude constant across all frequencies (Table 1). The magnitude was scaled so that SoS_13_ had a peak-to-peak amplitude of 1 deg. This yielded a 0.17°/s spectral velocity magnitude for each of the 5 frequency components. For the other trajectories, we maintained the magnitude of each spectral velocity component constant across all perturbation frequencies. This angular velocity magnitude was chosen based on Peterka (15) to make the task difficult while allowing participants to complete our test trials. This yielded peak-to-peak displacement amplitudes of 1.1051 deg. for the SoS_11_ signal, 0.9592 deg. for the SoS_15_ signal, and 0.8948 deg. for the SoS_17_ signal. These four trajectories each had the exact same velocity magnitude of 0.17 deg./s at each of their five disturbance frequencies (Figure 2B).

**Table 1:** The frequency, magnitude, and phase used to create each of the SoS time series are shown.

	Frequency (Hz)	Magnitude (deg)	$\emptyset_{o}$ (deg)	$\emptyset_{s}$ (deg)	Frequency (Hz)	Magnitude (deg)	$\emptyset_{o}$ (deg)	$\emptyset_{s}$ (deg)
	SoS_11_				SoS_13_			
f_1_	0.0604	0.4483	0	−90.75	0.0714	0.3793	0	72.27
f_2_	0.3241	0.0836	243	−112.8	0.3351	0.0808	230	−150.9
f_3_	0.5988	0.0452	184	−27.95	0.6207	0.0436	336	−115.8
f_4_	0.9833	0.0275	246	−52.52	0.9943	0.0272	333	−100.8
f_5_	1.3239	0.0205	90	−130.9	1.3788	0.0196	241	−163.6
	SoS_15_				SoS_17_			
f_1_	0.0824	0.3287	0	−96.00	0.0934	0.2900	0	−108.24
f_2_	0.3900	0.0694	110	−80.39	0.3680	0.0736	101	13.24
f_3_	0.7196	0.0376	112	−102.3	0.6976	0.0388	224	−55.18
f_4_	1.0602	0.0255	230	26.82	1.0492	0.0258	283	−128.37
f_5_	1.4447	0.0187	110	54.96	1.4117	0.0192	130	−23.94

$\emptyset_{o}$ represents the original phase value used to create the SoS signals. $\emptyset_{s}$ represents the shifted phase value calculated from the actual signal following a time-shift to start the steady-state of each of the SoS trajectories at a position zero-crossing.

We shifted the signals in time to begin motion at a displacement zero-crossing to minimize transients that otherwise might unduly perturb subjects at the beginning of the trial. Table 1 shows the original phase as well as the shifted phase values of SoS_11_, SoS_13_, SoS_15_, and SoS_17_ at each individual frequency.

We used a commercially available device (Virtualis MotionVR) to provide the platform tilt perturbations. The steady-state perturbation was designed to have a duration of 182.0444 s yielded by 16,384 (2^14^) points at the Virtualis platform’s sampling/refresh rate of 90 Hz. To allow the postural response to reach steady-state prior to the start of this steady-state cycle, 20s of stimuli identical to the last 20s of the steady state waveforms were added before the steady state waveform. We also added a 0.2 s ramp-up phase before the 20s steady state waveforms to minimize sudden motions that could potentially disturb our subjects or their responses. Since the steady-state cycle was repeated just once, the total perturbation duration was 202.2444 s for all trials. Supplementary Figure S1 shows example position and time trajectories. A custom Matlab script used to generate the waveforms is available on request.

### Test procedures

We collected postural response data from 24 healthy participants. A custom script written in Matlab first generated the SoS time series and the resultant CSV files were imported into the Virtualis MotionVR Research software to deliver platform perturbations. No passive head movements were applied, which leads platform perturbations to stimulate a human postural control system that includes vestibular, proprioceptive, and visual sensors. Center of pressure (CoP) data were collected by two embedded force plates at a sampling rate of 90 Hz.

All of the participants completed 6 trials per day on each of two separate days—yielding 12 trials in total for each subject (Table 2) (i.e., no dropouts). The two test days were separated by more than 1 day but less than 18 days. See Supplementary Figure S2 for more details. All but one participant completed the testing within 9 days. We randomized the order of the 12 trials to mitigate order effects. A different combination of SoS perturbation signals (e.g., SoS_11_ and SoS_13_) was used for each trial. Participants are allowed up to three attempts if an incomplete trial occurs (e.g., touching the safety rail around the Virtualis or taking a step). No incomplete trials occurred in this cohort, so all participants experienced each trial once. Participants’ feet were bare, with the outside edge of feet separated by 28 cm to mimic the standard Sensory Organization Test (SOT) conditions that are widely used in clinics. Arms were crossed with hands on shoulders. Participants were asked to close their eyes. Participants wore a virtual reality headset (HTC VIVE) that showed a dark screen to eliminate visual cues if a participant opened their eyes. Prior to testing, participants were instructed to stand as still as possible without any extra motions to avoid active movements (e.g., at the head, knee, and hip) that are not induced by platform perturbations or verbal responses. The VR headset has an IMU sensor that records 3D head linear positions and 3D head angular positions. Each participant was asked to rest at least 1 min between trials to minimize fatigue.

**Table 2:** The SoS trajectories selected for motion stimuli in each trial.

	Roll	Pitch	RALP	LARP
(i)-1	SoS_11_	SoS_15_	–	–
(i)-2	SoS_13_	SoS_17_	–	–
(i)-3	SoS_17_	SoS_11_	–	–
(i)-4	SoS_15_	SoS_13_	–	–
(ii)-1	–	–	SoS_13_	SoS_17_
(ii)-2	–	–	SoS_11_	SoS_15_
(ii)-3	–	–	SoS_15_	SoS_13_
(ii)-4	–	–	SoS_17_	SoS_11_
(iii)-1	SoS_11_	SoS_15_	SoS_13_	SoS_17_
(iii)-2	SoS_13_	SoS_17_	SoS_11_	SoS_15_
(iii)-3	SoS_17_	SoS_11_	SoS_15_	SoS_13_
(iii)-4	SoS_15_	SoS_13_	SoS_17_	SoS_11_

### Participants

The present study was approved by The Ohio State University Institutional Review Board. Prior to testing, all participants provided their written informed consent to participate in the study. Twenty-four healthy participants including 12 males and 12 females participated in the study. The age range of the participants was 21–65 yr (40.3 ± 13.1 years). To qualify as a healthy normal participant, strict criteria intended to eliminate conditions that might affect normal vestibular function or standing balance were assessed by a health questionnaire as a part of a central registry. These exclusion criteria included neurological disorders such as neurodegenerative disease, peripheral neuropathy, epilepsy, traumatic brain injury, or prior stroke; active vestibular disorders such as vestibular migraine, persistent postural-positional dizziness, Ménière disease, prior acute vestibular syndrome with peripheral vestibular loss, vestibular areflexia, or unresolved benign paroxysmal positional vertigo; orthopedic impairments affecting standing such as orthopedic injuries or impairments of the lower extremities; and active ongoing medical conditions (recent surgery, severe heart disease or pulmonary disease, and cancer).

### Postural analyses

We analyzed CoP data using Matlab R2024a. The first 20s of data for each trial were eliminated to remove the transient response at the onset of the perturbation. Each remaining CoP signal, having 16,384 data points representing 182.044 s was shifted to have zero mean and filtered using a zero-phase 2^nd^ order Butterworth low-pass filter with a cut-off frequency of 20 Hz.

To estimate RALP and LARP CoP sway, ML and AP CoP were rotated 45 degrees clockwise using the following equations.


$$\left[\begin{array}{c} \mathrm{Co}P_{\text{LARP}}\;(t)\\ \mathrm{Co}P_{\text{RALP}}\;(t) \end{array}]=[\begin{array}{cc} \cos45^{{}^{\circ}} & -\sin45^{{}^{\circ}}\\ \sin45^{{}^{\circ}} & \cos45^{{}^{\circ}} \end{array}][\begin{array}{c} \mathrm{Co}P_{\mathrm{ML}}\;(t)\\ \mathrm{Co}P_{\mathrm{AP}}\;(t) \end{array}\right]$$

A discrete Fourier transform (Matlab, fft.m) was applied to ML, AP, RALP, and LARP CoP signals and to roll, pitch, RALP and LARP perturbation signals. The complex numbers resulting from the discrete Fourier transform (DFT) were divided by the length of the data points (*N* = 16,384). The first half of the two-sided spectrum was removed, and the positive spectrum was multiplied by 2 to convert each two-sided amplitude spectrum to a single-sided amplitude spectrum. We used frequency response functions (FRF) to describe the sensitivity and phase of the CoP response relative to the tilt perturbation as a function of frequency. The FRF values are determined by dividing the complex DFT of the CoP by the complex DFT of the platform tilt angles ( $\mathrm{So}S_{11},\mathrm{So}S_{13},\mathrm{So}S_{15},\text{and}\;\mathrm{So}S_{17}$ ) at each of the five individual perturbation frequencies:


$$\begin{array}{l} \mathrm{FR}F_{\mathrm{ML}}(f_{k,i})=\frac{\mathrm{DFT}[\mathrm{Co}P_{\mathrm{ML}}(f_{k,i})]}{\mathrm{DFT}[\mathrm{So}S_{\text{Roll}}(f_{k,i})]}\;\\ \left(k=\mathrm{So}S_{11},\mathrm{So}S_{13},\mathrm{So}S_{15},\mathrm{So}S_{17})\;(\;i=1,2,\ldots 5\right) \end{array}$$

$$\begin{array}{l} \mathrm{FR}F_{\mathrm{AP}}(f_{k,i})=\frac{\mathrm{DFT}[\mathrm{Co}P_{\mathrm{AP}}(f_{k,i})]}{\mathrm{DFT}[\mathrm{So}S_{\text{Pitch}}(f_{k,i})]}\\ \;(k=\mathrm{So}S_{11},\mathrm{So}S_{13},\mathrm{So}S_{15},\mathrm{So}S_{17})\;(\;i=1,2,\ldots 5) \end{array}$$

$$\begin{array}{l} \mathrm{FR}F_{\text{RALP}}(f_{k,i})=\frac{\mathrm{DFT}[\mathrm{Co}P_{\text{RALP}}(f_{k,i})]}{\mathrm{DFT}[\mathrm{So}S_{\text{RALP}}(f_{k,i})]}\;\\ \left(k=\mathrm{So}S_{11},\mathrm{So}S_{13},\mathrm{So}S_{15},\mathrm{So}S_{17})\;(\;i=1,2,\ldots 5\right) \end{array}$$

$$\begin{array}{l} \mathrm{FR}F_{\text{LARP}}(f_{k,i})=\frac{\mathrm{DFT}[\mathrm{Co}P_{\text{LARP}}(f_{k,i})]}{\mathrm{DFT}[\mathrm{So}S_{\text{LARP}}(f_{k,i})]}\;\\ \left(k=\mathrm{So}S_{11},\mathrm{So}S_{13},\mathrm{So}S_{15},\mathrm{So}S_{17})\;(\;i=1,2,\ldots 5\right) \end{array}$$

We calculated the sensitivity (units of mm/degree) of the CoP postural response by calculating the magnitude of these FRF values at each frequency. Sensitivity is a non-negative value, and phase estimated via the frequency response functions is limited to a range between −180° and 180°. Phase values beyond $\pm 180^{{}^{\circ}}$ were calculated using the unwrap function in Matlab.

Additionally, we calculated time domain metrics—the root mean square distance (RMSD) and the mean velocity of the ML, AP, RALP, and LARP CoP in each of the 12 conditions [(i)1–4, (ii)1–4, and (iii)1–4]. The ML, AP, RALP, and LARP RMSD were calculated by taking the root mean square distance of the zero-mean, low-pass filtered CoP signals. The ML, AP, RALP, and LARP mean velocity were determined by dividing the total distance by the duration of the trial.

### Statistical analyses

For the time domain metrics – RMSD and mean velocity – we first performed eight sets of two-way repeated measures MANOVA to evaluate the effects of Condition (i, ii, iii) and Trial [e.g., (i)-1, (i)-2, (i)-3, and (i)-4] on the eight metrics that include RMSD and mean velocity in ML, AP, RALP, and LARP directions. Bonferroni-corrected pairwise comparisons were next performed to further examine differences across all 12 trials (3 conditions x 4 trials each). We then pooled the four trials within each condition and performed Bonferroni-corrected pairwise comparisons to evaluate condition-level differences.

For the spectral responses, Hotelling T-squared tests were used to determine whether spectral responses observed at perturbed frequencies were significantly different from zero. This helps demonstrate that those spectral responses are distinguishable from the remnant. We next performed four sets of four-way repeated measures MANOVA using real and imaginary components for ML, AP, RALP, and LARP frequency response functions (FRFs) that described the sensitivity and phase of CoP postural responses relative to the stimulus. For one-sample comparisons, we performed Hotelling T-squared tests using the real and imaginary parts of each FRF’s complex values. When comparing two different frequency sets, we calculated the 2-dimensional difference (i.e., the difference between real and the difference between imaginary components) between the two responses at each of the 5 perturbation frequencies. To address multiple comparisons, we applied both a commonly used Bonferroni correction to control the family-wise error rate by adjusting the significance level *α* from 0.05 to 0.05 divided by the number of comparisons, and the Benjamini-Hochberg (B-H) procedure to control the false discovery rate at q = 0.05, to mitigate the conservatism of the Bonferroni correction.

## Results

While the focus of this paper is on the frequency response functions (FRFs), we begin by presenting some traditional time-domain metrics and then present our primary frequency domain results. Supplementary Figure S3 shows exemplary raw traces of CoP ML and AP.

### Root mean square distance and mean velocity

Repeated measures MANOVA revealed significant effects of Condition [e.g., (i), (ii), and (iii)] and Trial [e.g., (i)-1, (i)-2, (i)-3, and (i)-4] on Root mean square distance (RMSD) (Condition: *p* < 0.001, Trial: *p* < 0.001) and mean velocity (Condition: *p* < 0.001, Trial: *p* = 0.038). We also found a significant interaction effect of Condition and Trial on RMSD (p < 0.001) but not mean velocity (*p* = 0.62). Supplementary Tables S1, S2 show *F*-values and *p*-values that indicate the significant main effects and interaction effects of Condition and Trial on RMSD and Mean Velocity.

**Figure 3:**
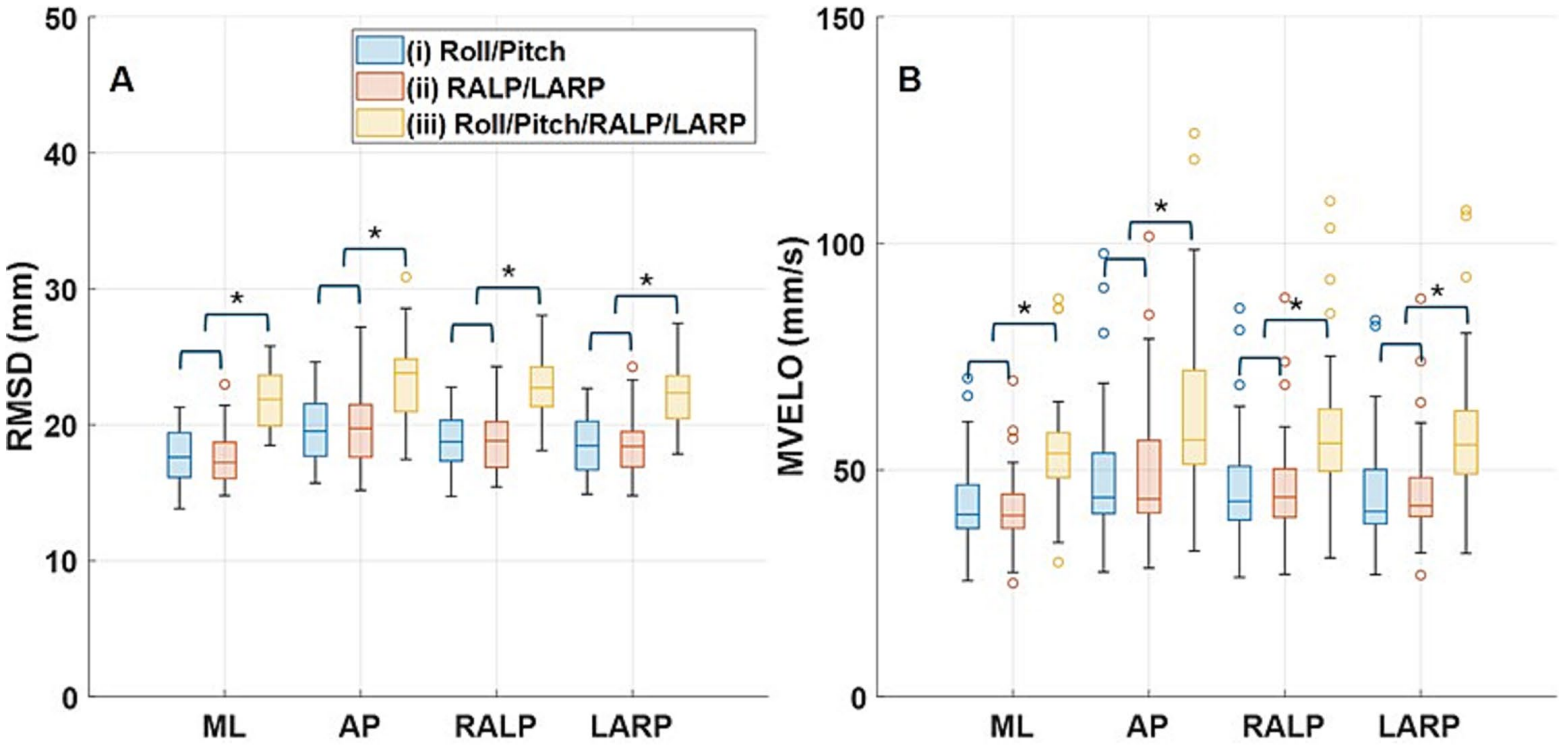
The box-and whisker plots show the distribution of RMSD (root mean square distance) **(A)** and MVELO (mean velocity) **(B)** across the 24 subjects. Boxes show 25th to 75th percentile. The horizontal line that splits the box shows the median and the lines that extend from the box show the range of the data. The dots that appear past the ends of whiskers represent outliers. The three colors delineate the three conditions. The asterisks delineate statistically significant differences (*p* < 0.05). Horizontal brackets show that across all RMSD and MVELO parameters, Condition (iii) was significantly different from Conditions (i) and (ii) (*p* < 0.0001), while Conditions (i) and (ii) were not significantly different from each other (*p* > 0.23).

Further Bonferroni corrected pairwise comparisons found a significant difference between Condition (i) vs. (iii) (*p* < 0.001) and Condition (ii) vs. (iii) (*p* < 0.001) for both RMSD and mean velocity, which is shown in Figure 3. The asterisks delineate statistically significant differences (*p* < 0.05). The pairwise comparisons showed no significant differences among the four trials in mean velocity but indicated a significant difference between Trial 1 vs. 3 in ML RMSD and Trial 1 vs. 4 in LARP RMSD but not one of the other 22 trial comparisons of RMSD. The peak-to-peak amplitudes for Condition (iii) are larger than those for Condition (i) and (ii), so it was expected that Condition (iii) would evoke larger postural responses than Condition (i) or (ii) in all the directions. Peak-to-peak displacement was roughly 50% greater for Condition (iii) (range: 1.53° to 2.09°) than for Condition (i) and Condition (ii) (range 0.89° to 1.40°); peak-to-peak amplitudes for Condition (i) and Condition (ii) were about the same. Supplementary Table S3 provides the peak-to-peak amplitudes of the stimuli delivered during the four trials in the three conditions.

Supplementary Figure S4 provides box-and whisker plots that show RMSD and mean velocity of the CoP responses for all of the subjects and for all the trials for each condition. We pooled the four trials in each condition as the differences between trials were inconsistent. Figure 3 shows box- and whisker plots of the average RMSD (Figure 3A) and mean velocity (Figure 3B) of ML, AP, RALP, and LARP CoP responses across the four trials for each condition [e.g., (i)-1, (i)-2, (i)-3, and (i)-4]. Bonferroni corrected pairwise comparisons, performed using a paired t-test, confirmed that both RMSD and mean velocity were significantly larger for Condition (iii) when roll, pitch, RALP, and LARP perturbations were provided simultaneously versus Condition (i) when just roll/pitch stimuli were provided and Condition (ii) when just RALP/LARP stimuli were provided in separate trials (*p* < 0.0001). No significant differences were observed when comparing RMSD or mean velocity responses evoked by roll/pitch stimuli [Condition (i)] to those evoked by RALP/LARP stimuli [Condition (ii)) (*p* > 0.23].

### Spectral responses

**Figure 4:**
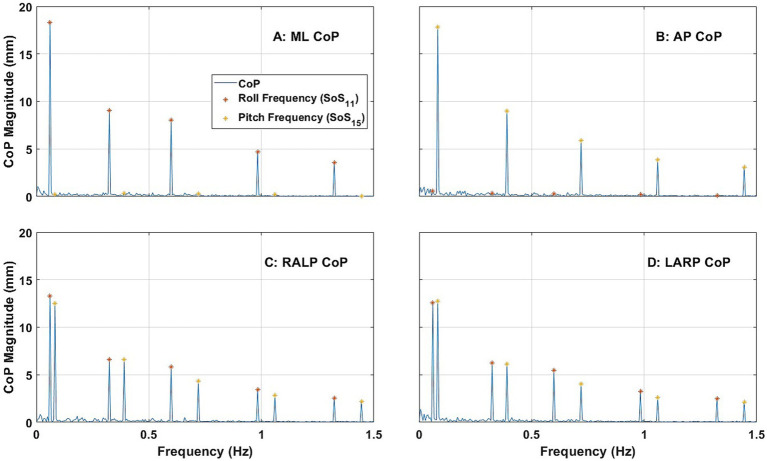
The power spectra plots show the average magnitude of the spectral response of CoP (center of pressure) in the ML (medial-lateral) **(A)**, AP (anterior–posterior) **(B)**, RALP (right anterior and left posterior) **(C)**, and LARP (left anterior and right posterior) **(D)** planes across the 24 subjects when SoS_11_ was provided for roll and SoS_13_ was provided for pitch [i.e., Condition (i)-1]. Different asterisk colors delineate CoP sway in response to roll (orange) and pitch (yellow) stimuli.

Figure 4 shows the average ML, AP, RALP, and LARP CoP sway spectral response across the 24 subjects for the roll/pitch condition (i)-1 (i.e., when provided SoS_11_ for roll and SoS_15_ for pitch). Consistent with previous literature (38), ML CoP spectral peaks were observed at each of the five frequencies for the roll tilt stimuli (Figure 4A); ML sway at each of the roll perturbation frequencies were the predominant response components. Similarly, AP CoP spectral peaks were observed at each of the five frequencies for the pitch tilt stimuli (Figure 4B); AP sway at each of the pitch perturbation frequencies were the predominant response components. These spectral peaks were clearly distinguishable from one another in the frequency domain. RALP and LARP CoP spectral magnitudes each show 10 response components during this roll/pitch condition. This is a direct outcome of the fact that the RALP/LARP coordinates are rotated 45 degrees from the roll/pitch coordinates, so RALP spectral peaks were observed at each of the five frequencies for both roll tilt stimuli and pitch tilt stimuli (Figure 4C); similarly, LARP spectral peaks were observed at each of the five frequencies for both roll tilt stimuli and pitch tilt stimuli (Figure 4D).

Hotelling T-squared tests found that the spectral peaks observed at each perturbation frequency (i.e., ML for roll stimuli, AP for pitch stimuli, RALP for roll and pitch stimuli, and LARP for roll and pitch stimuli) were significantly different than zero (*p* < 0.0001). See Supplementary Table S4 for more details.

**Figure 5:**
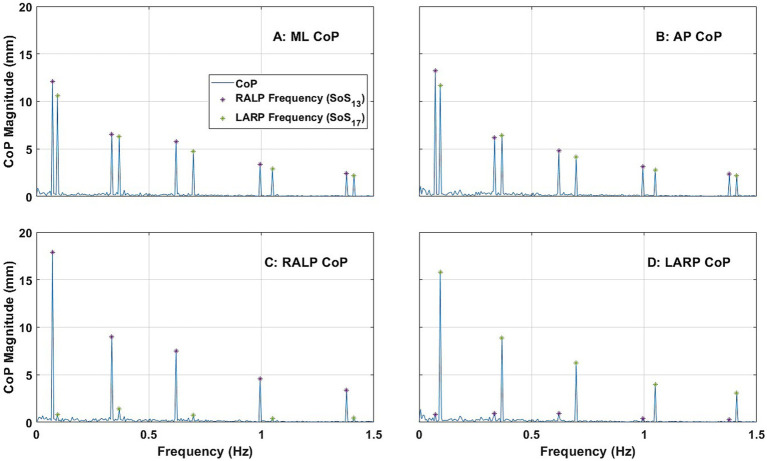
The power spectra plots show the average magnitude of the spectral response of CoP (center of pressure) in the ML (medial-lateral) **(A)**, AP (anterior–posterior) **(B)**, RALP (right anterior and left posterior) **(C)**, and LARP (left anterior and right posterior) **(D)** planes across the 24 subjects when SoS_13_ was provided for RALP and SoS_17_ was provided for LARP [i.e., Condition (ii)-1]. Different asterisk colors delineate CoP sway in response to RALP (purple) and LARP (green) stimuli.

Figure 5 shows the average ML, AP, RALP, and LARP CoP sway spectral magnitude across the 24 subjects for RALP/LARP condition (ii)-1 (i.e., when provided SoS_13_ for RALP and SoS_17_ for LARP). RALP spectral peaks were observed at each of the five frequencies for the RALP tilt stimuli (Figure 5C); sway in the RALP plane at each of the RALP perturbation frequencies were the predominant response components (Figure 5C). LARP spectral peaks were observed at each of the five frequencies for the LARP tilt stimuli (Figure 5D); sway in the LARP plane at each of the LARP perturbation frequencies were the predominant response components (Figure 5D). The RALP and LARP spectral peaks were distinguishable from one another. Analogous to the RALP and LARP responses shown above (Figure 4) for roll and pitch stimuli, ML (Figure 5A) and AP (Figure 5B) CoP spectral magnitudes each show 10 response components—one for each of the RALP and LARP perturbation frequencies.

Although the primary focus of the study was CoP measurements, Supplementary Figure S5 shows spectral head RALP and LARP response components at RALP and LARP frequencies. RALP head response components were statistically larger than LARP head response components at all RALP frequencies (*p* < 0.0001); LARP head response components were statistically larger than RALP head response components at all LARP frequencies (*p* < 0.0001). This suggests that RALP/LARP platform perturbations evoke head motions in the perturbed directions.

Hotelling T-squared tests found that the spectral peaks observed at each perturbation frequency (i.e., ML for RALP and LARP stimuli, AP for RALP and LARP stimuli, RALP for RALP stimuli, and LARP for LARP stimuli) were significantly different than zero (*p* < 0.0001). See Supplementary Table S4 for more details.

**Figure 6:**
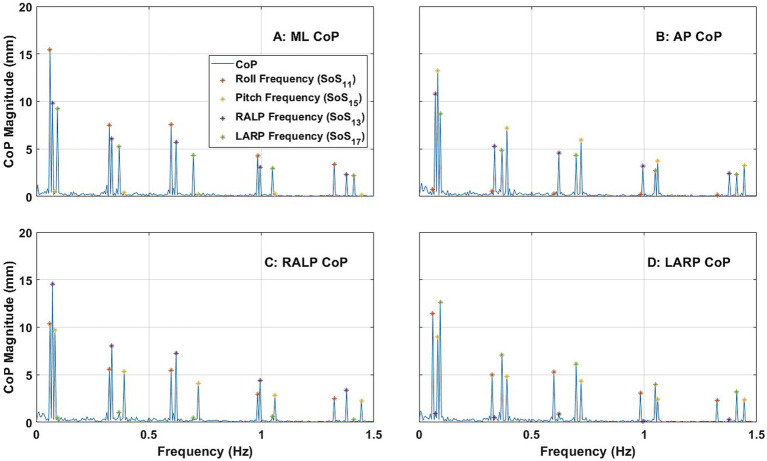
The power spectra plots show the average magnitude of the spectral response of CoP (center of pressure) in the ML (medial-lateral) **(A)**, AP (anterior–posterior) **(B)**, RALP (right anterior and left posterior) **(C)**, and LARP (left anterior and right posterior) **(D)** planes across the 24 subjects when SoS_11_ was provided for roll and SoS_15_ was provided for pitch, SoS_13_ was provided for RALP, and SoS_17_ was provided for LARP [i.e., Condition (iii)-1]. Different asterisk colors delineate CoP sway in response to roll (orange), pitch (yellow), RALP (purple), and LARP (green) stimuli.

Figure 6 shows the average ML, AP, RALP, and LARP CoP sway spectral magnitude across the 24 subjects for RALP/LARP condition (iii)-1 (i.e., when SoS_11_ was provided for roll, SoS_15_ was provided for pitch, SoS_13_ was provided for RALP, and SoS_17_ was provided for LARP). ML spectral peaks were observed at each of the roll, RALP, and LARP perturbation frequencies (Figure 6A); AP spectral peaks were observed at each of the pitch, RALP, and LARP perturbation frequencies (Figure 6B). Similarly, RALP spectral peaks were observed at each of the RALP, roll, and pitch perturbation frequencies (Figure 6C); LARP spectral peaks were observed at each of the LARP, roll, and pitch perturbation frequencies (Figure 6D). Each CoP spectral magnitude shows 15 response components at three sets of five perturbation frequencies in the directions described above. This demonstrates that spectral postural response components were observed at each perturbation frequency—except for responses that were spatially orthogonal to the stimuli (e.g., AP responses to roll stimuli or RALP responses to LARP stimuli)—when four SoS perturbations are provided in roll, pitch, RALP, and LARP directions simultaneously.

Hotelling T-squared tests found that the spectral peaks observed at each perturbation frequency (i.e., ML for roll, RALP, and LARP stimuli, AP for pitch, RALP, and LARP stimuli, RALP for RALP, roll, and pitch stimuli, and LARP for LARP, roll, and pitch stimuli) were significantly different than zero (*p* < 0.0001). See Supplementary Table S4 for more details.

### Sensitivity and phase

**Figure 7:**
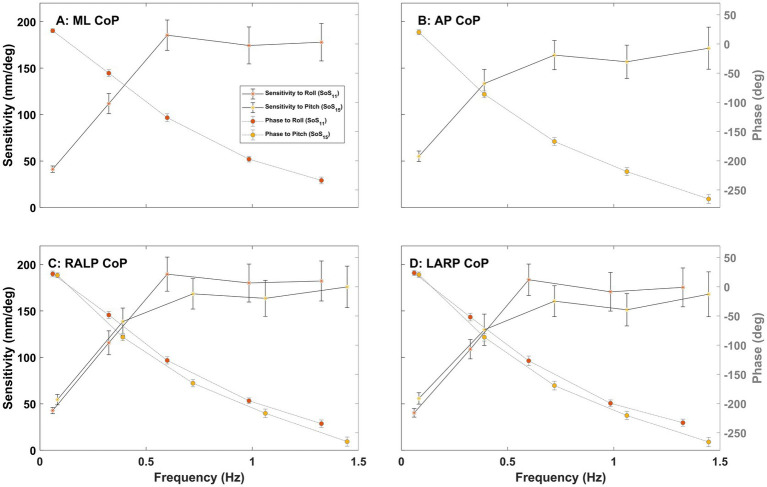
The plots show the sensitivity (left y-axis) and phase (right y-axis) of the CoP (center of pressure) response in the ML (medial-lateral) **(A)**, AP (anterior–posterior) **(B)**, RALP (right anterior and left posterior) **(C)**, and LARP (left anterior and right posterior) **(D)** planes versus perturbation stimulus frequency for Condition (i) with SoS_11_ provided for roll and SoS_15_ provided for pitch. Error bars show 95% confidence intervals. Different asterisk colors delineate the average sensitivity of CoP sway in response to roll (orange) and pitch (yellow) stimuli.

**Figure 8:**
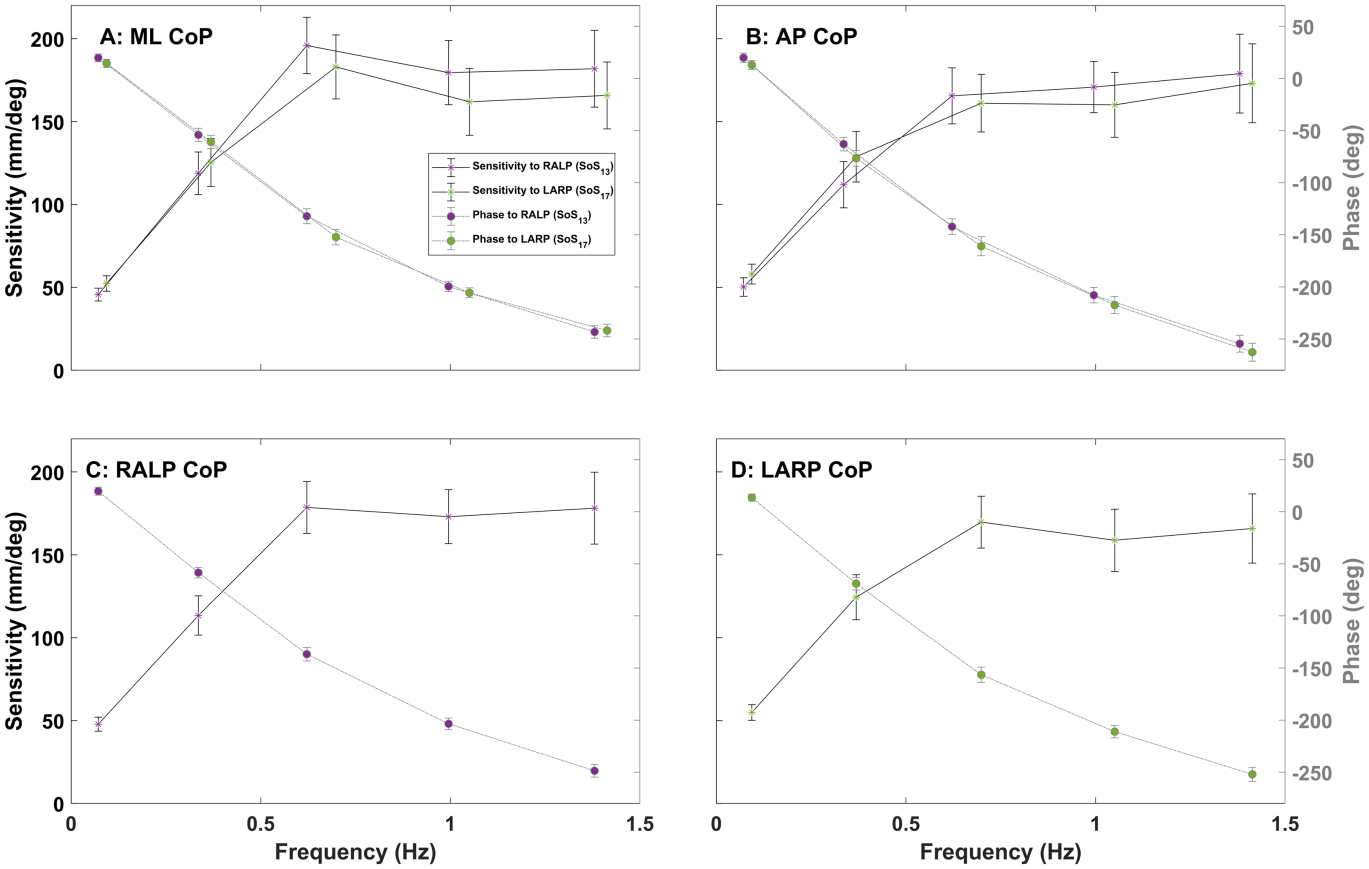
The plots show the sensitivity (left y-axis) and phase (right y-axis) of the CoP (center of pressure) response in the ML (medial-lateral) **(A)**, AP (anterior–posterior) **(B)**, RALP (right anterior and left posterior) **(C)**, and LARP (left anterior and right posterior) **(D)** planes versus perturbation stimuli frequency for Condition (ii) with SoS_13_ provided for RALP and SoS_17_ provided for LARP. Error bars show 95% confidence intervals. Different asterisk colors delineate the average sensitivity of CoP sway in response to RALP (purple) and LARP (green) stimuli.

Figures 7, 8 show the sensitivity (left, y-axis) and phase (right, y-axis) of the CoP spectral response relative to the perturbation stimuli in the ML, AP, RALP, and LARP planes. Consistent with earlier reports (15), (1) the sensitivity increased with increasing perturbation frequency for the low to mid-frequency range (0.06–0.70 Hz) and reached a plateau for the high-frequency range (0.70–1.42 Hz), and (2) phase lag increased with increasing perturbation frequency. The sensitivity of CoP RALP and LARP in response to roll stimuli is a little lower than the sensitivity in response to pitch stimuli at the low-frequency range (0.06–0.50 Hz) but a little higher at the mid to high-frequency range (0.50–1.42 Hz) (Figures 7C,D). The sensitivity of CoP ML and AP in response to RALP stimuli is slightly lower than the sensitivity in response to LARP stimuli in the low-frequency range (0.06–0.50 Hz) but slightly higher in the mid to high-frequency range (0.50–1.42 Hz) (Figures 8A,B). Supplementary Figure S6 shows the sensitivity and phase plots for Condition (iii).

The real and imaginary components of ML, AP, RALP, and LARP spectral responses were evaluated using a four-way repeated measures MANOVA to find a difference when the stimuli were provided in roll/pitch coordinates versus RALP/LARP coordinates. The four factors were (1) the three stimulus directions that were not orthogonal to the response direction (e.g., roll, RALP, and LARP tilts, but not pitch tilt, for the ML response), (2) the four sets of perturbation frequencies (i.e., SoS_11_, SoS_13_, SoS_15_, and SoS_17_), (3) the five frequencies for each set (i.e., f_1_, f_2_, f_3_, f_4_, and f_5_), and (4) the three test conditions (i.e., roll/pitch only, RALP/LARP only, and simultaneous roll/pitch + RALP/LARP conditions). Mauchly’s test indicated the sphericity assumption was invalid. While some statistically significant differences were observed, a clear pattern of differences was not evident. See Supplementary Table S5 for details. That some differences were statistically significant, alongside violation of the sphericity assumption, encouraged additional statistical evaluation of these data using more refined analyses, Hotelling’s T-squared analyses with Bonferroni correction and the Benjamini-Hochberg (BH) procedure, which follow.

### Roll/pitch stimuli and RALP/LARP stimuli evoke similar AP, ML, RALP, and LARP responses

#### Simultaneous roll/pitch/RALP/LARP stimuli (160 comparisons)

**Figure 9:**
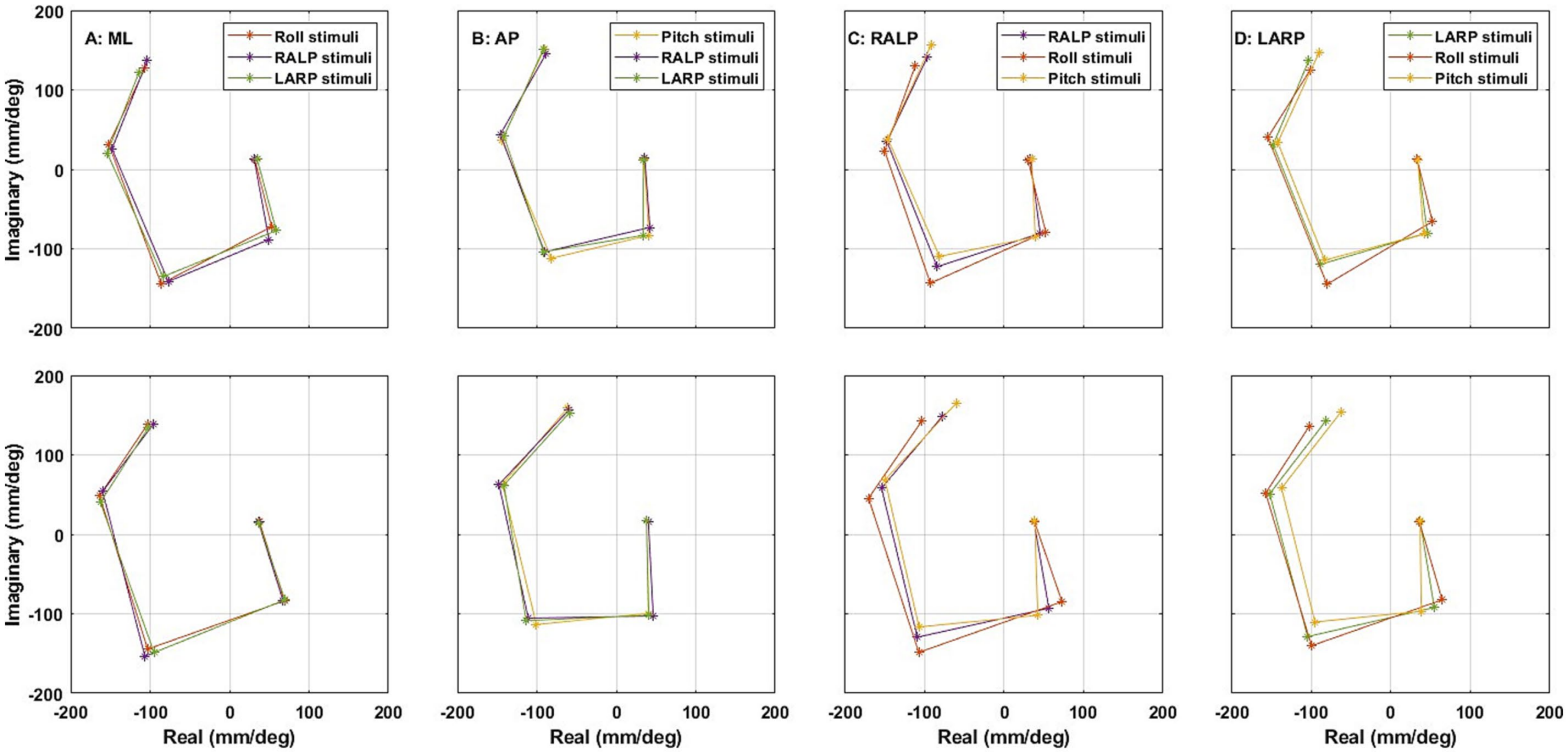
The average frequency response functions of CoP (center of pressure) **(A)** ML (medial-lateral), **(B)** AP (anterior–posterior), **(C)** RALP (right anterior and left posterior), and **(D)** LARP (left anterior and right posterior) in the complex plane across the 24 subjects (top) when simultaneous roll/pitch/RALP/LARP stimuli were provided using SoS_11_ [i.e., Condition (iii)] and (bottom) when roll/pitch stimuli [i.e., Condition (i)] were provided using SoS_11_ on separate trials than RALP/LARP stimuli [i.e., Condition (ii)]. Different asterisk colors delineate the average frequency responses to roll (orange), pitch (yellow), RALP (purple), and LARP (green) stimuli. All plots spiraled clockwise with increasing frequency. See Supplementary Figures S7–S9 for more details using SoS_13_, SoS_15_, and SoS_17_.

The top panels of Figure 9 show the average frequency response functions in the complex plane across the 24 healthy subjects when the simultaneous roll/pitch/RALP/LARP stimuli [i.e., Condition (iii)] were provided. The radius of the complex number ( $r=\sqrt{a^{2}+b^{2}}$ for the complex number is a+bi ) represents the sensitivity (mm/deg) of the postural response relative to the stimulus. The angle of the complex number represents the phase ( ∅=atan2(b,a) ), where atan2 is the 4-quadrant arc tangent of the postural response relative to the stimulus. The frequency response functions spiral clockwise with increasing perturbation frequency. These plots show the real and imaginary components of the responses, which will be used for the Hotelling’s T-squared analyses that follow. Specifically, we performed a Hotelling’s T-squared test to compare:

- ML sway evoked by roll stimuli *versus* RALP stimuli,
- ML sway evoked by roll stimuli *versus* LARP stimuli,
- AP sway evoked by pitch stimuli *versus* RALP stimuli,
- AP sway evoked by pitch stimuli *versus* LARP stimuli,
- RALP sway evoked by RALP stimuli *versus* roll stimuli,
- RALP sway evoked by RALP stimuli *versus* pitch stimuli,
- LARP sway evoked by LARP stimuli *versus* roll stimuli, and
- LARP sway evoked by LARP stimuli *versus* pitch stimuli.

After calculating the discrete Fourier transform, we took the vector difference between the real and imaginary components of the two spectra (e.g., normalized ML sway in response to roll stimuli minus normalized ML sway in response to RALP stimuli) at each stimulation frequency for each participant. The above 8 conditions (a through h) x 4 SoS signals x 5 frequencies yielded 160 Hotelling’s T-squared comparisons in total. Without correcting for multiple comparisons, we found 26 significant differences (*p* < 0.05). Bonferroni correction and the Benjamini-Hochberg (BH) procedure found no significant difference between any of these 160 postural response components. To provide context for later results, Table 3 shows the 10 lowest uncorrected *p*-values and the associated BH critical values.

**Table 3:** The 10 lowest *p*-values of 160 comparisons (a) through (h) during simultaneous roll/pitch/RALP/LARP stimuli [i.e., Condition (iii)] are listed.

	*p*-value	BH	Response	Stimuli	Frequency	Set
1	0.0024	0.0003	RALP	(f) RALP vs. pitch	f_1_	SoS_13_
2	0.0063	0.0006	RALP	(f) RALP vs. pitch	f_3_	SoS_13_
3	0.0078	0.0009	LARP	(h) LARP vs. pitch	f_2_	SoS_13_
4	0.0079	0.0013	RALP	(f) RALP vs. pitch	f_5_	SoS_13_
5	0.0088	0.0016	LARP	(g) LARP vs. roll	f_2_	SoS_11_
6	0.0112	0.0019	AP	(c) pitch vs. RALP	f_2_	SoS_11_
7	0.0117	0.0022	RALP	(f) RALP vs. pitch	f_4_	SoS_15_
8	0.0142	0.0025	AP	(d) pitch vs. LARP	f_1_	SoS_17_
9	0.0154	0.0028	LARP	(g) LARP vs. roll	f_2_	SoS_15_
10	0.0169	0.0031	ML	(b) roll vs. LARP	f_2_	SoS_13_

By definition, the familiar, though conservative, Bonferroni correction equals the first BH value, which is 0.0003 for this analysis. For context, response direction analyzed, stimuli compared, frequency, and SoS test set are also listed.

Stated differently, when roll/pitch/RALP/LARP stimuli were provided simultaneously, we found: (1) that ML postural responses to roll stimuli were not significantly different from ML responses to RALP or LARP stimuli, (2) that AP postural responses to pitch stimuli were not significantly different from AP responses to RALP or LARP stimuli, (3) that RALP postural responses to RALP stimuli were not significantly different from RALP responses to roll or pitch stimuli, and (4) that LARP postural responses to LARP stimuli were not significantly different from LARP responses to roll or pitch stimuli.

#### Roll/pitch stimuli and RALP/LARP stimuli provided on separate trials (160 comparisons)

The bottom panels of Figure 9 show the average frequency response functions in the complex plane across the 24 healthy subjects when the 2D roll/pitch stimuli [i.e., Condition (i)] were provided on separate trials than the 2D RALP/LARP stimuli (i.e., Condition (ii)). The frequency response functions again spiral clockwise with increasing perturbation frequency. Our Hotelling’s T-squared analyses of these data mimicked the analyses presented in the previous section where the roll, pitch, RALP, and LARP stimuli were all presented simultaneously. Hotelling’s T-squared testing yielded 31 significant differences (*p* < 0.05) out of 160 comparisons. A Bonferroni correction and the BH procedure each yield one (by definition, the same) significant difference—the LARP response to roll stimuli yielded a significantly different LARP response than evoked by the LARP stimuli at 1.4465 Hz (f_5_) for the SoS_15_ (*p* < 0.0003). We explicitly note that not one of the other three frequency sets (i.e., SoS_11_, SoS_13_, and SoS_17_) showed a significant difference for this same condition following the BH procedure. To provide context for later results, Table 4 shows the 10 lowest uncorrected p-values and the associated BH critical values. The five lowest p-values were all found at f_5_, the highest frequency.

**Table 4:** The 10 lowest *p*-values of 160 comparisons (a) through (h) when the roll/pitch stimuli [i.e., Condition (i)] and the RALP/LARP stimuli [i.e., Condition (ii)] were provided on separate trials.

	*p*-value	BH	Response	Stimuli	Frequency	Set
1	0.0001	0.0003	LARP	(g) LARP vs. roll	f_5_	SoS_15_
2	0.0014	0.0006	LARP	(h) LARP vs. pitch	f_5_	SoS_17_
3	0.0017	0.0009	LARP	(g) LARP vs. roll	f_5_	SoS_13_
4	0.0025	0.0013	RALP	(f) RALP vs. pitch	f_5_	SoS_13_
5	0.0026	0.0016	RALP	(e) RALP vs. roll	f_5_	SoS_11_
6	0.0027	0.0019	LARP	(h) LARP vs. pitch	f_2_	SoS_15_
7	0.0051	0.0023	LARP	(g) LARP vs. roll	f_5_	SoS_11_
8	0.0055	0.0025	LARP	(h) LARP vs. pitch	f_2_	SoS_11_
9	0.0055	0.0028	RALP	(f) RALP vs. pitch	f_2_	SoS_15_
10	0.0061	0.0031	RALP	(e) RALP vs. roll	f_2_	SoS_15_

By definition, the familiar, though conservative, Bonferroni correction equals the first BH value, which is 0.0003 for this analysis. For context, response direction analyzed, stimuli compared, frequency, and SoS test set are also listed.

In summary, these results (i.e., when the 2D roll/pitch stimuli were provided on different trials than the 2D RALP/LARP stimuli) appear similar to results when roll/pitch/RALP/LARP stimuli were provided simultaneously. Specifically, we found that ML, AP, RALP, and LARP postural responses were not significantly different when the 2D stimuli were provided in roll/pitch coordinates versus RALP/LARP coordinates.

### Small frequency shifts associated with our different SoS frequency sets yield significant differences in postural responses

**Figure 10:**
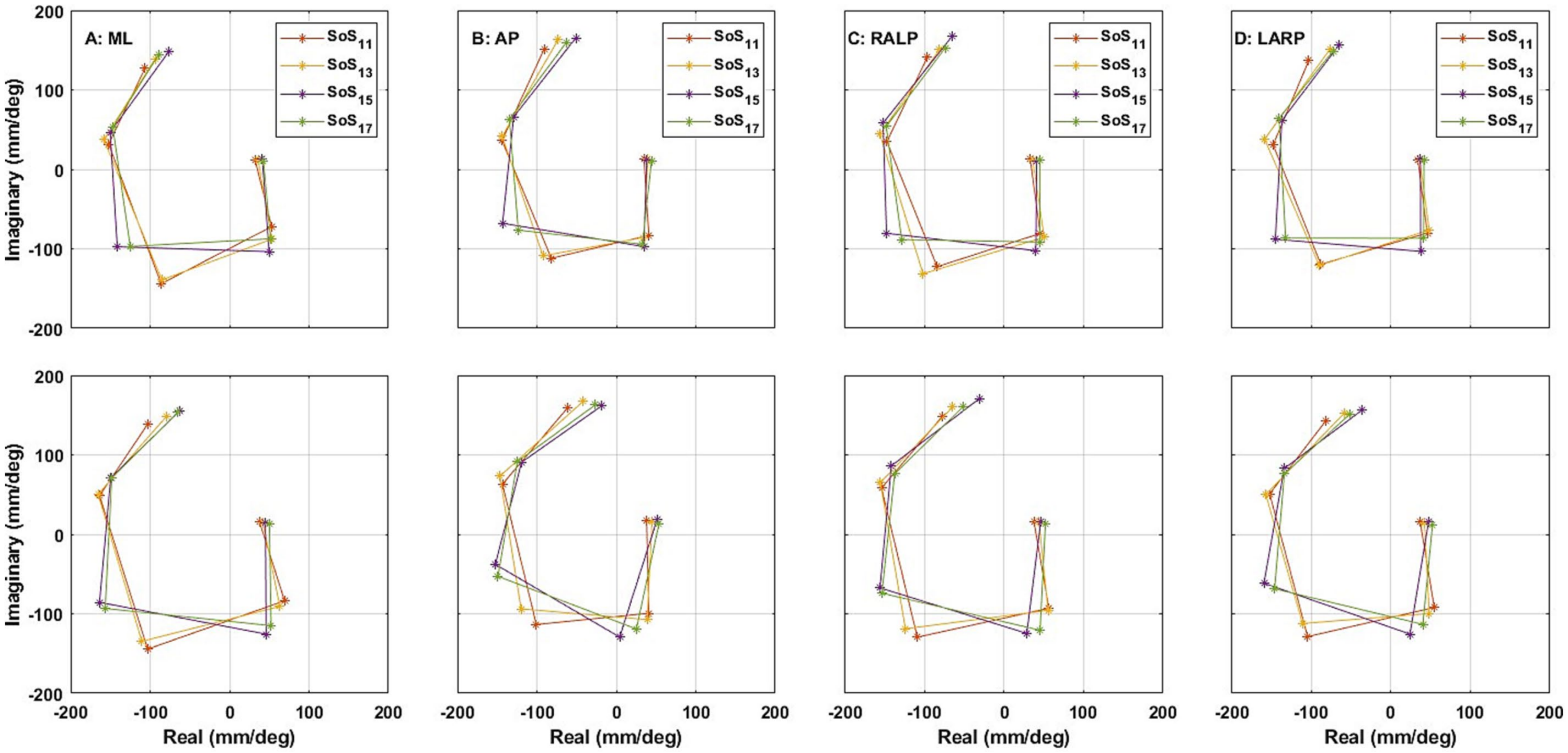
The average frequency response functions of CoP (center of pressure) **(A)** ML (medial-lateral), **(B)** AP (anterior–posterior), **(C)** RALP (right anterior and left posterior), and **(D)** LARP (left anterior and right posterior) in the complex plane across the 24 subjects (top) when simultaneous roll/pitch/RALP/LARP stimuli were provided [i.e., Condition (iii)] and (bottom) when roll/pitch stimuli [i.e., Condition (i)] were provided on separate trials than RALP/LARP stimuli [i.e., Condition (ii)]. Different asterisk colors delineate the average frequency responses to SoS_11_ (orange), SoS_13_ (yellow), SoS_15_ (purple), and SoS_17_ (green) stimuli. All plots spiraled clockwise with increasing frequency.

Earlier publications, as well as our data (Figures 7, 8), clearly establish that responses to balance perturbations vary systematically with frequency (11, 15, 38). Nevertheless, to help establish the sensitivity of these methods to small frequency changes and to tell if we can directly compare responses at nearby individual frequencies across our four different stimuli sets (SoS_11_, SoS_13_, SoS_15_, SoS_17_), we evaluated the impacts of small frequency changes on the postural responses utilizing the four SoS signals described in detail in the methods (Figure 2). Using formatting similar to Figure 9, Figure 10 shows the average frequency response functions of ML, AP, RALP and LARP CoP sway in the complex plane at the five perturbation frequencies for SoS_11_, SoS_13_, SoS_15_, and SoS_17_ when provided as: (i) roll/pitch only, (ii) RALP/LARP only, and (iii) roll/pitch/RALP/LARP. The data demonstrates distinct separations for the different sets of the five perturbation frequencies.

Analogous to earlier analyses, 240 comparisons were performed using Hotelling’s T-squared test to investigate how those four specific frequency sets (SoS_11_, SoS _13_, SoS _15_, and SoS _17_) and their five frequencies (f_1_ through f_5_) affect ML, AP, RALP, and LARP CoP sway. After calculating the discrete Fourier transform, we took the vector difference between the real and imaginary components of the two spectra for each stimulation frequency for each participant and performed a Hotelling’s T-square test to compare CoP in response to (aa) SoS_11_ and SoS_13_, (bb) SoS_11_ and SoS_15_, (cc) SoS_11_ and SoS_17_, (dd) SoS_13_ and SoS_15_, (ee) SoS_13_ and SoS_17_, and (ff) SoS_15_ and SoS_17_. This yielded 60 comparisons (each of 6 SoS stimuli comparisons x each of 5 frequencies x each of 2 response directions) for (i) roll/pitch only, 60 comparisons for (ii) RALP/LARP only, and 120 comparisons for (iii) roll/pitch/RALP/LARP (each of 6 SoS stimuli comparisons x each of 5 frequencies x each of 4 response directions), which means 240 comparisons in total. Hotelling’s T-squared test found 168 significant differences. Bonferroni correction yielded 75 significant differences, which is 31.25% of the *p*-values, and the B-H procedure yielded 145 significant differences, which is 60.41% of the p-values. Table 5 shows the 10 lowest original p-values and the individual p-values’ BH critical values. Specifically, we found that small frequency shifts between (aa) SoS_11_ and SoS_13_, (bb) SoS_11_ and SoS_15_, (cc) SoS_11_ and SoS_17_, (dd) SoS_13_ and SoS_15_, (ee) SoS_13_ and SoS_17_, and (ff) SoS_15_ and SoS_17_ yielded significant differences in postural responses.

**Table 5:** The 10 lowest *p*-values of 240 comparisons between different frequency sets (aa)–(ff).

	*p*-value	BH	Condition	Response	Frequency	Frequency set	Δf(Hz)
1	<0.0001	0.0002	(ii)	LARP	f_5_	(bb) SoS_11_ vs. SoS_15_	0.1208
2	<0.0001	0.0004	(i)	AP	f_2_	(bb) SoS_11_ vs. SoS_15_	0.0659
3	<0.0001	0.0006	(iii)	RALP	f_3_	(dd) SoS_13_ vs. SoS_15_	0.0989
4	<0.0001	0.0008	(ii)	LARP	f_3_	(bb) SoS_11_ vs. SoS_15_	0.1208
5	<0.0001	0.0010	(i)	AP	f_2_	(dd) SoS_13_ vs. SoS_15_	0.0549
6	<0.0001	0.0013	(i)	ML	f_1_	(cc) SoS_11_ vs. SoS_17_	0.0330
7	<0.0001	0.0015	(ii)	LARP	f_1_	(cc) SoS_11_ vs. SoS_17_	0.0330
8	<0.0001	0.0017	(iii)	ML	f_3_	(bb) SoS_11_ vs. SoS_15_	0.1208
9	<0.0001	0.0019	(iii)	AP	f_3_	(bb) SoS_11_ vs. SoS_15_	0.1208
10	<0.0001	0.0021	(ii)	LARP	f_3_	(dd) SoS_13_ vs. SoS_15_	0.0989

Stated briefly, when comparing responses at distinct frequencies from our four perturbation trajectories that were separated by 0.0110–0.1208 Hz (i.e., relatively small differences in perturbation frequencies), we found statistically significant differences for the majority of these comparisons. Since we already knew that postural responses vary with frequency, the finding that the majority of comparisons yielded significant differences helps establish the sensitivity of these Hotelling’s T-squared methods to detect even subtle differences associated with small changes in perturbation frequency, which bolsters the absence of significant differences reported above in the section entitled “Roll/Pitch stimuli and RALP/LARP stimuli evoke similar AP, ML, RALP, and LARP responses.”

Future studies with larger sample sizes could help verify these findings and improve the reliability and precision of effect size estimates.

## Discussion

In this study, we recruited 24 healthy participants to measure changes in the CoP evoked by pseudorandom SoS platform perturbations to characterize reactive balance in three conditions. In Condition (i), we provided two distinct balance perturbation stimuli that were orthogonal in both time and space in the roll and pitch tilt motion dimensions. In Condition (ii), we provided two distinct balance perturbation stimuli that were orthogonal in both time and space in the RALP and LARP tilt motion dimensions defined by the anatomy of the semicircular canals (Figure 1). In Condition (iii), we provided four distinct balance perturbation stimuli that were orthogonal in time (but not space) in the roll, pitch, RALP, and LARP tilt motion dimensions. We were able to quantify AP and ML responses even when the perturbation stimuli were provided in RALP/LARP coordinates [i.e., Condition (ii)]. The AP and ML responses were not significantly different from those evoked by the perturbation stimuli provided in roll/pitch coordinates. These primary findings demonstrate the potential of the platform perturbations provided in RALP/LARP coordinates as a new balance assessment that will ultimately help us observe the balance impairment of individuals with asymmetric sensory deficits in the vertical semicircular canals.

### Spectral response components

Consistent with our earlier findings (38), we report that: (a) tilt perturbations in the roll plane yielded responses primarily in the ML direction (Figure 5A) with only small responses in the AP direction (Figure 5A), and (b) tilt perturbations in the pitch plane yielded responses primarily in the AP direction (Figure 5B) with only small responses in the ML direction (Figure 5B). As novel findings, we report that (c) tilt perturbations in the RALP plane yielded responses primarily in the RALP direction (Figure 6C; Supplementary Figure S5) with only small responses in the LARP direction (Figure 6C; Supplementary Figure S5), and (d) tilt perturbations in the LARP plane yielded responses primarily in the LARP direction (Figure 6D; Supplementary Figure S5) with only small responses in the RALP direction (Figure 6D; Supplementary Figure S5).

In Condition (i), where we provided simultaneous roll and pitch stimuli, we found that the largest response components were in the ML and AP directions (Figures 5A,B). When we look at these same data in coordinates that align with the RALP and LARP directions, we (by the mathematical relationship between these two different reference frames) found response components that reflect the projection of the roll and pitch responses onto the RALP and LARP directions (Figures 5C,D).

In Condition (ii), where we provided simultaneous RALP and LARP stimuli, we found that the largest response components were in the RALP and LARP directions (Figures 6C,D). Again, because of the mathematical nature of reference frames that are rotated with respect to one another, when we look at these same data in ML and AP coordinates, we found response components that reflect the projection of the predominant RALP and LARP responses onto the ML and AP directions (Figures 6A,B).

In Condition (iii), where we provided simultaneous stimuli in each of 4 planes (i.e., roll, pitch, RALP, and LARP)—each having 5 different stimulus frequencies, we found frequency response components at all 20 frequencies. Clear response components were found for: (a) roll, RALP, and LARP for each frequency of the roll stimuli, (b) pitch, RALP, and LARP for each frequency of the pitch stimuli, (c) roll, pitch, and RALP for each frequency of the RALP stimuli, and (d) roll, pitch, and LARP for each frequency of the LARP stimuli. In other words, clear response components were found at each stimulation frequency except that clear consistent response components were not found in the response direction that was spatially orthogonal to the stimulus direction.

### No significant differences in ML and AP responses between roll/pitch stimuli and RALP/LARP stimuli

The repeated measures MANOVA using real and imaginary components found that the ML and AP responses were not significantly different when the tilt stimuli were provided in roll/pitch coordinates vs. RALP/LARP coordinates [Pillai’s Trace, AP: *p* = 0.48, ML: *p* = 0.63]. Similarly, Hotelling’s T-square for further pairwise comparisons found no significant differences in the sensitivity of ML and AP spectral responses between roll and pitch tilt stimuli versus RALP and LARP tilt stimuli after the Bonferroni correction and the Benjamini-Hochberg (BH) procedure. This was true when the roll and pitch stimuli were provided in different trials (i.e., Condition (i)) than the RALP and LARP stimuli [i.e., Condition (ii)] and when the roll, pitch, RALP, and LARP stimuli were provided simultaneously in the same trial [i.e., Condition (iii)]. This suggests that we can compare ML and AP responses evoked by RALP and LARP stimuli to existing literature showing ML and AP responses evoked by roll and pitch stimuli. The central nervous system is known to integrate the signals from the three semicircular canals to estimate 3D angular velocity and 2D tilt (46, 47). While speculative, these multidimensional estimates that arise via sensorimotor integration may be used for postural control via ML and AP motor templates (34, 35).

Putting all of the above into context, this suggests that we can provide tilt stimuli in the RALP and LARP coordinates and:

1. Quantify the ML and AP responses evoked by RALP and LARP tilt stimuli, making it straightforward to compare with – and build on – the pre-existing literature that typically quantifies ML and AP responses evoked by 1D roll or 1D pitch stimuli.
2. Quantify the responses in the RALP/LARP coordinates, making it easier to separate contributions of the RALP semicircular canals from the LARP canals.

For example, if a patient has a lesion in the right anterior semicircular canals, the patient will show a larger postural response to RALP tilt stimuli than to LARP tilt stimuli due to the limited contribution of the right anterior semicircular canals. We anticipate that 2D balance perturbations in roll/pitch coordinates are less likely to distinguish balance impairment due to asymmetric sensory deficits in the vertical semicircular canals (i.e., RALP and LARP) as all of the four vertical semicircular canals contribute to postural control at the same time when perturbations are provided in roll or pitch coordinates.

Furthermore, in the future, we intend to provide translation stimuli in the AP and ML directions while we simultaneously provide tilt stimuli in the RALP and LARP directions. Consistent with the approach shown herein, these four stimuli will all be orthogonal in time and spectrally separable. Nonetheless, providing the tilt stimuli in RALP/LARP directions and translation in the more traditional ML and AP directions adds spatial separation alongside the orthogonality in time and spectral separation mentioned earlier.

### Frequency response functions

The plots of the sensitivity and phase of the CoP spectral responses (Figures 7, 8) are also qualitatively similar to earlier studies that provided pseudorandom pitch tilt stimuli with eyes closed (15) and roll tilt stimuli with eyes closed (20). Prior research generated support surface pitch tilts using a pseudorandom ternary sequence (PRTS) and quantified center of mass (CoM) AP body sway angles of 8 healthy subjects using a backboard (15). They found (1) that transfer function gain increased in the low-mid frequency range (0.0165–0.5 Hz) and decreased in the higher frequency range (0.5–2.48 Hz) and (2) that phase lag continuously increased with frequency (15). Another prior study estimated CoM ML body sway angles using a backboard and demonstrated that transfer function gain increased with frequency below 0.5 Hz and phase lag continuously increased with frequency in a bandwidth of 0.021–2.79 Hz in response to roll support surface PRTS tilts (20). When we estimate FRFs for the CoM by low-pass filtering CoP data (48) with a cut-off frequency of 0.47 Hz, our data (see Supplementary Figures S10–S12 for more details) demonstrate FRFs that mimic those from two earlier studies (15, 20).

### Small changes in frequency matter

We created four pseudorandom SoS trajectories that were designed to have frequency components that were close to each other (Table 1). Even though we were aware (see previous section) that frequency response functions varied with frequency (15), we hypothesized that the small frequency differences we designed would not yield significant response differences because the frequencies were near to one another (e.g., SoS_11_ f_1_ = 0.0604 Hz and SoS_17_ f_1_ = 0.0934 Hz) and because the response variability between participants would be too large to yield significant differences. However, the four-way repeated measures MANOVA showed a significant effect of the four pseudorandom SoS trajectories (i.e., SoS_11_, SoS_13_, SoS_15_, and SoS_17_) on the responses (*p* < 0.001). The Hotelling’s T-square for further pairwise comparisons also indicated that those four trajectories evoked significantly different responses (Table 5). This suggests to us we should not compare frequency response functions (FRFs) at different perturbation frequencies (e.g., f_1_ of SoS_11_ versus f_1_ of SoS_13_) as small changes in frequency matter for these FRFs. We were pleased by this unexpected finding because this (inadvertently) shows the power of the approach presented herein, where statistically significant differences in the postural response were found for nearby stimulation frequencies; this shows that response variability had to have been small enough to unveil the response differences evoked by small differences in the stimulus frequency. This would not have occurred if the methods did not yield clean spectral response components. The high precision in spectral CoP responses also implies complexity when comparing directly across studies unless selected perturbation frequencies are exactly identical. Modeling using transfer functions (15) will come into play to fit the experimental data to address this limitation.

### Some technical considerations

Positive integers only were used to multiply the fundamental frequency to determine the frequency of all stimulus components. This means that complete cycles for each frequency are provided in each full period of stimulation. This makes the stimuli provided at each frequency orthogonal in time over the full steady-state period. Both theoretically and practically, this means that no stimulus frequency components interfere with any other stimulus frequency components. Theoretically, the same holds true for steady-state responses of any linear system, as is commonly assumed by many studies of the balance responses evoked by 1D balance perturbations (12, 20, 25–33).

We chose a smaller fundamental frequency (0.0055 Hz) than the one selected (0.044 Hz) in our previous study (38) to allow all perturbation frequencies to fit in our target bandwidth of 0.05–2.00 Hz. This bandwidth was selected because early literature showed that vestibular thresholds decreased with increasing frequency in the bandwidth (49). To define our frequency components, we multiplied the fundamental frequency of 0.0055 Hz by prime numbers with the single exception of 15; 15 was included, since its prime factors of 3 and 5 were not included as multipliers. This set of multipliers – like sets of prime number multipliers – reduces the chance of system nonlinearities yielding overlapping response frequency components.

We chose to begin with 11 as the lowest multiplier. This meant that a minimum of 11 cycles of stimuli were provided for each complete fundamental period of stimulation. This is above the minimum rule of 5 to 7 cycles often required for spectral analyses (15).

Choosing 11 as the lowest multiplier also allowed us to also include 13, 15, and 17 as the lowest integer multipliers, which leads to the fundamental frequency of the four trajectories used herein being separated only by a factor of 1.55. In other words, there was an increase of 55% from the lowest fundamental frequency of 0.060 Hz to the highest fundamental frequency of 0.093 Hz-meaning that these 4 stimuli all had similar—but separable—fundamental frequencies (see Figure 2; Table 1).

To accomplish an analogous goal, two pseudorandom ternary sequence (PRTS) stimuli with non-overlapping harmonics have been interleaved by doubling the fundamental frequency of the 2nd stimuli (e.g., a 100% increase) (22). Taking this approach for four stimuli—like those provided in our Condition (iii) would result in the fourth fundamental frequency (i.e., SoS_17_ f_1_) being 8 times greater than the first fundamental frequency (i.e., SoS_11_ f_1_) (i.e., a 700% increase). For 12 stimuli (e.g., 6 physical motion dimensions and 6 visual motion dimensions), which we may consider testing in the future, this approach of doubling the fundamental frequency for each motion dimension stimulated would mean that the highest fundamental frequency would be 2,048 times greater (i.e., 2^(12–1)^, which is more than 3 orders of magnitude greater) than the lowest fundamental frequency—almost certainly putting either the lowest or the highest fundamental frequency outside the physiologic range of interest.

We chose to perform our statistics using the real and imaginary Cartesian-coordinate response components instead of the more traditional polar coordinate response components of magnitude and phase. Standard coordinate transformations were used to convert back-and-forth from the standard Cartesian coordinates (i.e., real and imaginary components) and standard polar coordinates (i.e., magnitude and phase). The Cartesian coordinates offer the advantage that these two independent variables are each unbounded (i.e., range from minus ∞ to plus ∞). For comparison, magnitude is bounded at zero (i.e., is non-negative) and phase of the Fourier transform is bounded to fall in a range of −180 to 180°. As we discovered, these bounds alter statistical calculations even though the data points being compared are identical. While phase “unwrapping” can yield a greater phase range as the function shifts the angles to make the jump between consecutive angles less than 180° the unwrapping process is always subject to assumptions, which are avoided by simply using the real and imaginary components. Furthermore, the units on the real and imaginary variables are always the same, which means the weighting in all calculations—including, but not limited to, statistical calculations—is also naturally the same.

### Limitations

While the present study represents the potential of two-dimensional balance perturbations in canal coordinates (i.e., RALP and LARP), it does not demonstrate the contributions of the RALP and LARP semicircular canals to postural control. Future studies will need to show whether individuals with peripheral lesions in a specific vertical semicircular canal (i.e., impaired perception of RALP motion cues) show balance impairment in response to perturbations delivered in the same plane. That will demonstrate the potential of our pseudorandom balance perturbations provided in RALP/LARP coordinates to characterize how peripheral lesions impact postural control.

Our data sets did not allow us to determine the motor control strategies that led to the observed postural responses as we did not measure muscle activations using electromyography (EMG). Additional studies are required to determine whether the motor control system disaggregates the sensory signals provided in RALP/LARP coordinates into roll/pitch coordinates to sway in ML and AP directions or the motor control activates muscles in RALP/LARP coordinates when provided perturbations in RALP/LARP coordinates. We suspect that a mixture of different muscle synergies (34, 35, 50) (i.e., hip and ankle strategies (51–53)) are involved in responding to our two-dimensional balance perturbations delivered in RALP/LARP coordinates.

We identified some additional limitations of the study:

1. As the two force plates embedded in the motion platform record only 2D CoP data (i.e., ML and AP), we were not able to analyze force or torque data. As just one example, if the force plates provided signals representing the three forces in x-y-z coordinates, it would allow us to determine the ground reaction force, which plays a key role in estimating spatial orientation using proprioceptive cues at the feet.
2. We did not collect postural response data at the hip to better estimate body sway angle near the Center of Mass (CoM). Future studies will consider using a motion tracker at the hip to enhance the ability to characterize responses at more anatomical landmarks.
3. As we delivered perturbations in RALP/LARP coordinates, our primary interest was the vestibular modality in the canals. Thus, this study did not consider the interactions of vestibular and proprioceptive cues.
4. The CoP data were not evenly sampled from the age of 21–65 in 24 participants since the goal of the study was to evaluate the new technology using two-dimensional platform perturbations. We are in the process of collecting normative data from the age of 18–89 in over 100 participants to identify the effect of age on postural control by using a comprehensive test protocol that includes perturbed balance assessments, but this data set is unavailable at this time.
5. Although the trial order was randomized and at least a 1-min break was provided for each participant, six trials a day may lead to learning or fatigue effects. Data from a separate study that we hope to publish soon showed that multidimensional platform perturbations demonstrated excellent repeatability (Intraclass correlations (ICC) > 0.88) for four repetitions with a 1-min break in between, which suggests that while learning or fatigue effects are real, they may not be major concerns.
6. Our perturbation paradigm involves technical complexity for reproduction in non-specialist laboratories or clinics. One of our long-term goals of the paradigm is to license this novel technology to balance platform manufacturers, and we are willing to share the code that we have generated to create the motion trajectories used herein.
7. This study included only healthy adults, so we were not able to demonstrate the potential of our two-dimensional balance assessment using perturbations delivered in RALP/LARP coordinates for clinical use. But we share a scenario that we can envision. Imagine a patient showing up in a general balance clinic with disorientation and imbalance. If the patient screens with a significantly larger postural response to RALP tilts but not to LARP tilts compared to a normal range, this observation might suggest a semicircular canal deficit in the RALP plane. The result could lead clinicians to request more focused specialty testing (e.g., head impulse testing) and could thereby lead to the patient obtaining the appropriate intervention to compensate for their sensory deficit. Additional studies should further develop this comprehensive multisensory multidimensional balance assessment test (CoMMBAT) to investigate whether it can distinguish individuals with sensory deficits in vertical semicircular canals (e.g., vestibular hypofunction), peripheral neuropathy, and/or those with movement disorders (e.g., Parkinson’s disease or stroke) to demonstrate clinical applicability.

Finally, generally, we hypothesize that balance patient populations will show a larger postural response than healthy individuals. This would suggest that we should explore multidimensional balance assessments such as three-dimensional perturbations that add earth-vertical translations to the RALP and LARP tilt stimuli used for the present study. The earth-vertical perturbations might evoke a larger postural response in individuals with movement disorders than those with sensory deficits in the vertical semicircular canals as it would be hard for those with movement disorders to generate corrective torques using muscles to counteract gravitational torques to enhance their stability in response to the external perturbations.

## Conclusion

The present study demonstrated (1) that spectral response components were observed at each perturbed frequency when we provided two distinct balance perturbation stimuli in roll/pitch coordinates or/and RALP/LARP coordinates, (2) that the AP and ML postural responses were not significantly different when the stimuli were provided in roll/pitch coordinates versus RALP/LARP coordinates except for one of 320 comparisons as described in the results, (3) that even small shifts in perturbation frequency impact quantification of spectral postural responses, and (4) that spectral response components were observed at each of the 20 perturbation frequencies when SoS perturbations were provided in the four directions (roll, pitch, RALP, and LARP) simultaneously. These findings highlight that healthy individuals show similar postural sway in response to roll/pitch stimuli that are orthogonal to one another in both space and time, and RALP/LARP stimuli that are similarly orthogonal to one another in both space and time. Our two-dimensional balance assessment that provides platform perturbations in the canal coordinates might have the potential to contribute to the identification of a canal deficit that causes balance impairment. The anatomically driven novel approach seems poised to advance the field of neuro-otology.

## Data Availability

The raw data supporting the conclusions of this article will be made available by the authors, without undue reservation.

## References

[1] Nashner LM Black FO Wall C. Adaptation to altered support and visual conditions during stance: patients with vestibular deficits. J Neurosci. (1982) 2:536–44. doi: 10.1523/JNEUROSCI.02-05-00536.1982, PMID: 6978930 PMC6564270

[2] Perucca L Robecchi Majnardi A Frau S Scarano S. Normative data for the neurocom® sensory organization test in subjects aged 80–89 years. Front Hum Neurosci. (2021) 15:262. doi: 10.3389/FNHUM.2021.761262 PMC8641293 34867246

[3] Peterka RJ Black FO. Age-related changes in human posture control: motor coordination tests. J Vestib Res. (1990) 1:87–96. doi: 10.3233/ves-1990-1109, PMID: 1670140

[4] Cohen HS Kimball KT. Usefulness of some current balance tests for identifying individuals with disequilibrium due to vestibular impairments. J Vestib Res-Equilib Orientat. (2008) 18:295–303. doi: 10.3233/VES-2008-185-606 PMC2819299 19542603

[5] Tang P Moore S. Correlation between two clinical balance measures in older adults: functional mobility and sensory organization test. J Gerontol A. (1998) 53:140–6. 10.1093/gerona/53a.2.m140 9520921

[6] Campbell KR King LA Parrington L Fino PC Antonellis P Peterka RJ. Central sensorimotor integration assessment reveals deficits in standing balance control in people with chronic mild traumatic brain injury. Front Neurol. (2022) 13:897454. doi: 10.3389/FNEUR.2022.897454, PMID: 36341095 PMC9634071

[7] Wagner AR Merfeld DM. A modified two-dimensional sensory organization test that assesses both anteroposterior and mediolateral postural control. Front Rehabil Sci. (2023) 4:1166859. doi: 10.3389/FRESC.2023.1166859, PMID: 37284337 PMC10239846

[8] Franz JR Francis CA Allen MS O’Connor SM Thelen DG. Advanced age brings a greater reliance on visual feedback to maintain balance during walking. Hum Mov Sci. (2015) 40:381–92. doi: 10.1016/J.HUMOV.2015.01.012, PMID: 25687664 PMC4372858

[9] Franz JR Francis CA Allen MS Thelen DG. Visuomotor entrainment and the frequency-dependent response of walking balance to perturbations. IEEE Trans Neural Syst Rehabil Eng. (2017) 25:1135–42. doi: 10.1109/TNSRE.2016.2603340, PMID: 28113592 PMC5623133

[10] Kazanski ME Cusumano JP Dingwell JB. How healthy older adults regulate lateral foot placement while walking in laterally destabilizing environments. J Biomech. (2020) 104:109714. doi: 10.1016/J.JBIOMECH.2020.109714, PMID: 32139095 PMC7188576

[11] Maki BE Holliday PJ Topper AK. A prospective study of postural balance and risk of falling in an ambulatory and independent elderly population. J Gerontol. (1994) 49:M72–84. doi: 10.1093/GERONJ/49.2.M72, PMID: 8126355

[12] McAndrew PM Dingwell JB Wilken JM. Walking variability during continuous pseudo-random oscillations of the support surface and visual field. J Biomech. (2010) 43:1470–5. doi: 10.1016/J.JBIOMECH.2010.02.003, PMID: 20346453 PMC2866814

[13] Sinitksi EH Terry K Wilken JM Dingwell JB. Effects of perturbation magnitude on dynamic stability when walking in destabilizing environments. J Biomech. (2012) 45:2084–91. doi: 10.1016/J.JBIOMECH.2012.05.039, PMID: 22749389 PMC9128720

[14] Joseph Jilk D Safavynia SA Ting LH. Contribution of vision to postural behaviors during continuous support-surface translations. Exp Brain Res. (2014) 232:169–80. doi: 10.1007/S00221-013-3729-4/FIGURES/6, PMID: 24132526 PMC4065169

[15] Peterka RJ. Sensorimotor integration in human postural control. J Neurophysiol. (2002) 88:1097–118. doi: 10.1152/JN.2002.88.3.1097, PMID: 12205132

[16] Peterka RJ Statler KD Wrisley DM Horak FB. Postural compensation for unilateral vestibular loss. Front Neurol. (2011) 2:57. doi: 10.3389/FNEUR.2011.00057 21922014 PMC3167354

[17] Wiesmeier IK Dalin D Maurer C. Elderly use proprioception rather than visual and vestibular cues for postural motor control. Front Aging Neurosci. (2015) 7:124510. doi: 10.3389/FNAGI.2015.00097 PMC4477145 26157386

[18] Zoetbrood E. Alfred S Ronald V Bert-Jan B. The influence of the velocity of support surface rotations on sensory reweighting during standing balance. (2020).

[19] Assländer L. Sensory reweighting dynamics in human postural control. J Physiol Org. (2014) 111:1852–64. doi: 10.1152/jn.00669.2013, PMID: 24501263 PMC4044370

[20] Chvatal M Peterka RJ. Stimulus-dependent changes in the vestibular contribution to human postural control. J Neurophysiol. (2006) 95:2733–50. doi: 10.1152/JN.00856.2004 16467429

[21] Lippi V Assländer L Akcay E Mergner T. Body sway responses to pseudorandom support surface translations of vestibular loss subjects resemble those of vestibular able subjects. Neurosci Lett. (2020) 736:135271. doi: 10.1016/J.NEULET.2020.135271, PMID: 32710917

[22] Lippi V Maurer C Mergner T. Human body-sway steady-state responses to small amplitude tilts and translations of the support surface—effects of superposition of the two stimuli. Gait Posture. (2023) 100:139–48. doi: 10.1016/J.GAITPOST.2022.12.003, PMID: 36521258

[23] Pasma JH Engelhart D Maier AB Schouten AC Van Der Kooij H Meskers CGM. Changes in sensory reweighting of proprioceptive information during standing balance with age and disease. J Neurophysiol. (2015) 114:3220–33. doi: 10.1152/JN.00414.2015, PMID: 26424578 PMC4686291

[24] van Kordelaar J Pasma JH Cenciarini M Schouten AC van der Kooij H Maurer C. The reliance on vestibular information during standing balance control decreases with severity of vestibular dysfunction. Front Neurol. (2018) 9:351014. doi: 10.3389/FNEUR.2018.00371, /BIBTEX PMC5994722 29915556

[25] Grneberg C Duysens J Honegger F Allum JHJ. Spatio-temporal separation of roll and pitch balance-correcting commands in humans. J Neurophysiol. (2005) 94:3143–58. doi: 10.1152/JN.00538.2004, PMID: 16033938

[26] Jeka J Kiemel T Creath R Horak F Peterka R. Controlling human upright posture: velocity information is more accurate than position or acceleration. J Neurophysiol. (2004) 92:2368–79. doi: 10.1152/JN.00983.2003 15140910

[27] Mohebbi A Amiri P Kearney RE. Identification of human balance control responses to visual inputs using virtual reality. J Neurophysiol. (2022) 127:1159–70. doi: 10.1152/JN.00283.2021, PMID: 35353629

[28] Payne AM Hajcak G Ting LH. Dissociation of muscle and cortical response scaling to balance perturbation acceleration. J Neurophysiol. (2019) 121:867–80. doi: 10.1152/JN.00237.2018, PMID: 30517039 PMC6520627

[29] Payne AM Ting LH. Balance perturbation-evoked cortical N1 responses are larger when stepping and not influenced by motor planning. J Neurophysiol. (2020) 124:1875–84. doi: 10.1152/JN.00341.2020, PMID: 33052770 PMC7814905

[30] Welch T. A feedback model explains the differential scaling of human postural responses to perturbation acceleration and velocity. J Neurophysiol. (2009) 101:3294–309. doi: 10.1152/jn.90775.2008, PMID: 19357335 PMC2694108

[31] Willaert J Martino G Desloovere K Van Campenhout A Ting LH De Groote F. Increased muscle responses to balance perturbations in children with cerebral palsy can be explained by increased sensitivity to center of mass movement. Gait Posture. (2024) 107:121–9. doi: 10.1016/J.GAITPOST.2023.03.014, PMID: 36990910 PMC10517062

[32] Meyer P Oddsson L. Reduced plantar sensitivity alters postural responses to lateral perturbations of balance. Spring. (2004) 157:526–36. doi: 10.1007/s00221-004-1868-3, PMID: 15029466

[33] Rietdyk S. Patla A. Winter D.,. Balance recovery from medio-lateral perturbations of the upper body during standing. Elsevier. (1999). Available online at: https://www.sciencedirect.com/science/article/pii/S0021929099001165 (Accessed March 5, 2025). 10.1016/s0021-9290(99)00116-5 10541064

[34] Chvatal SA Torres-Oviedo G Safavynia SA Ting LH. Common muscle synergies for control of center of mass and force in nonstepping and stepping postural behaviors. J Neurophysiol. (2011) 106:999–1015. doi: 10.1152/JN.00549.2010, PMID: 21653725 PMC3154805

[35] Torres-Oviedo G Ting LH. Muscle synergies characterizing human postural responses. J Neurophysiol. (2007) 98:2144–56. doi: 10.1152/JN.01360.2006, PMID: 17652413

[36] Massion J. Postural control Systems in Developmental Perspective. Neurosci Biobehav Rev. (1998) 22:465–72. doi: 10.1016/S0149-7634(97)00031-6, PMID: 9595556

[37] Kailath T. Linear systems. Englewood Cliffs, NJ: Prentice-Hall (1980).

[38] Wagner AR Chirumbole SG Caccese JB Chaudhari AMW Merfeld DM. Development and validation of a two-dimensional pseudorandom balance perturbation test. Front Hum Neurosci. (2024) 18:1132. doi: 10.3389/FNHUM.2024.1471132 PMC11659295 39713174

[39] Allum JHJ Oude Nijhuis LB Carpenter MG. Differences in coding provided by proprioceptive and vestibular sensory signals may contribute to lateral instability in vestibular loss subjects. Exp Brain Res. (2008) 184:391–410. doi: 10.1007/S00221-007-1112-Z, PMID: 17849108

[40] Bloem B Allum JHJ Carpenter M Verschuuren J Honegger F. Triggering of balance corrections and compensatory strategies in a patient with total leg proprioceptive loss. Exp Brain Res. (2002) 142:91–107. doi: 10.1007/S00221-001-0926-3, PMID: 11797087

[41] Carpenter MG Allum JHJ Honegger F. Directional sensitivity of stretch reflexes and balance corrections for normal subjects in the roll and pitch planes. Exp Brain Res. (1999) 129:93–113. doi: 10.1007/S002210050940, PMID: 10550507

[42] Carpenter MG Allum JHJ Honegger F. Vestibular influences on human postural control in combinations of pitch and roll planes reveal differences in spatiotemporal processing. Exp Brain Res. (2001) 140:95–111. doi: 10.1007/S002210100802 /METRICS, PMID: 11500802

[43] Torres-Oviedo G Macpherson JM Ting LH. Muscle synergy organization is robust across a variety of postural perturbations. J Neurophysiol. (2006) 96:1530–46. doi: 10.1152/JN.00810.2005, PMID: 16775203

[44] MacDougall HG Weber KP McGarvie LA Halmagyi GM Curthoys IS. The video head impulse test: diagnostic accuracy in peripheral vestibulopathy. Neurology. (2009) 73:1134–41. doi: 10.1212/WNL.0B013E3181BACF85, PMID: 19805730 PMC2890997

[45] Wolfe J. M. Sensation & perception. 22. (2025). Available online at: https://global.oup.com/academic/product/sensation-and-perception-9780197663813 (accessed May 1, 2024).

[46] Clark TK Newman MC Karmali F Oman CM Merfeld DM. Mathematical models for dynamic, multisensory spatial orientation perception. Prog Brain Res. (2019) 248:65–90. doi: 10.1016/BS.PBR.2019.04.014, PMID: 31239146

[47] Merfeld DM Zupan L Peterka RJ. Humans use internal models to estimate gravity and linear acceleration. Nature. (1999) 398:615–8. doi: 10.1038/19303, PMID: 10217143

[48] Peterka RJ Murchison CF Parrington L Fino PC King LA. Implementation of a central sensorimotor integration test for characterization of human balance control during stance. Front Neurol. (2018) 9:413696. doi: 10.3389/FNEUR.2018.01045 PMC6300494 30619027

[49] Lim K Karmali F Nicoucar K Merfeld DM Karmali LK Merfeld NK. Perceptual precision of passive body tilt is consistent with statistically optimal cue integration. J Neurophysiol. (2017) 117:2037–52. doi: 10.1152/jn.00073.2016 28179477 PMC5434481

[50] Ting LH McKay JL. Neuromechanics of muscle synergies for posture and movement. Curr Opin Neurobiol. (2007) 17:622–8. doi: 10.1016/J.CONB.2008.01.002, PMID: 18304801 PMC4350235

[51] Creath R Kiemel T Horak F Peterka R Jeka J. A unified view of quiet and perturbed stance: simultaneous co-existing excitable modes. Neurosci Lett. (2005) 377:75–80. doi: 10.1016/j.neulet.2004.11.071, PMID: 15740840

[52] Horak FB Nashner LM. Central programming of postural movements: adaptation to altered support-surface configurations. J Neurophysiol. (1986) 55:1369–81. doi: 10.1152/JN.1986.55.6.1369, PMID: 3734861

[53] Horak FB Macpherson JM. Postural orientation and equilibrium In: . Handbook of physiology. Exercise: Regulation and integration of multiple systems. Neural control of movement. Bethesda, MD: American Physiology Society (1996). 255–92.

