# Supplementary material for: Anterior–posterior and medial-lateral balance metrics are unchanged when two-dimensional pseudorandom motion perturbations are provided in semicircular canal coordinates
Source: Front Neurol. 2025 Aug 25;16:1638493. doi: 10.3389/fneur.2025.1638493 (PMC12414766; doi:10.3389/fneur.2025.1638493)
Supplement: Supplementary file 1 [file Data_Sheet_1.PDF]

Supplementary

Methods

Generation of the Sum-of-Sines (SoS) Signals

As we needed to simultaneously provide four distinct balance perturbation stimuli in Condition (iii), we generated four steady-state Sum of Sinusoid (SoS) displacement trajectories using these three equations below in addition to the equation stated in the methods.

$$SoS_{13}(t) = \sum_{i=1}^{5} A_{13,i} \sin\left(2\pi f_{SoS13,i} t + \emptyset_{SoS13,i}\right)$$

$$SoS_{15}(t) = \sum_{i=1}^{5} A_{15,i} \sin\left(2\pi f_{SoS15,i} t + \emptyset_{SoS15,i}\right)$$

$$SoS_{17}(t) = \sum_{i=1}^{5} A_{17,i} \sin\left(2\pi f_{SoS17,i} t + \emptyset_{SoS17,i}\right)$$

In these equations, $A_{13,i}, A_{15,i}, and\ A_{17,i}$ are the i'th amplitudes $f_{13,i}, f_{15,i}, and\ f_{17,i}$ are the i'th frequencies, and $\emptyset_{13,i}, \emptyset_{15,i}, and\ \emptyset_{17,i}$ are the i'th phase values. **Figure S1** shows (A) the displacement and (B) the velocity of the four trajectories in time domain.

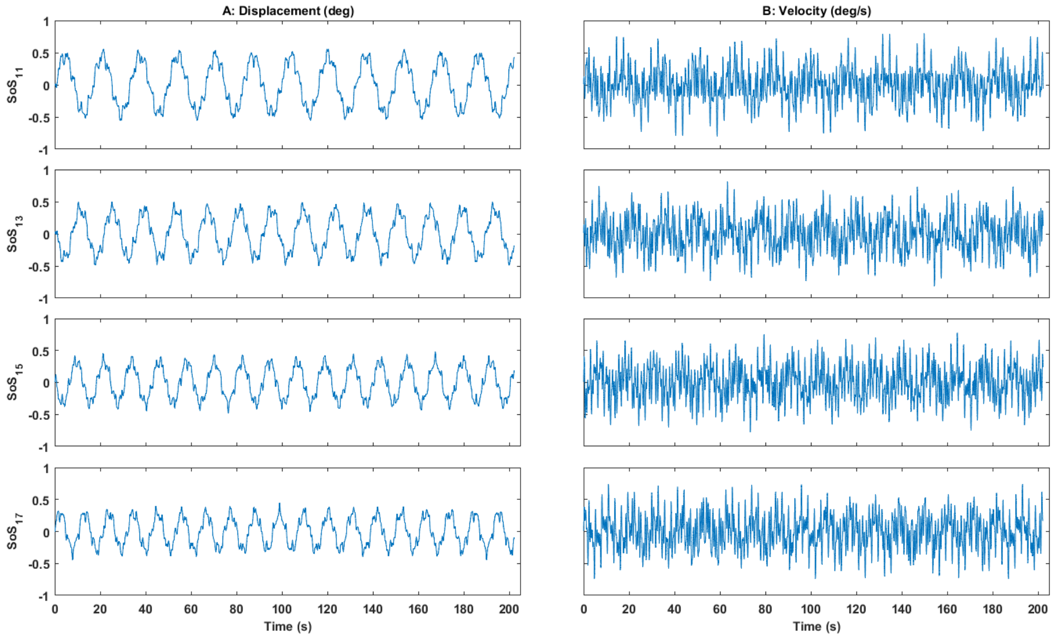

Figure. S1 The displacement (A) and velocity (B) of the SoS_{11}, SoS_{13}, SoS_{15}, and SoS_{17} trajectories over time.

Test Procedures

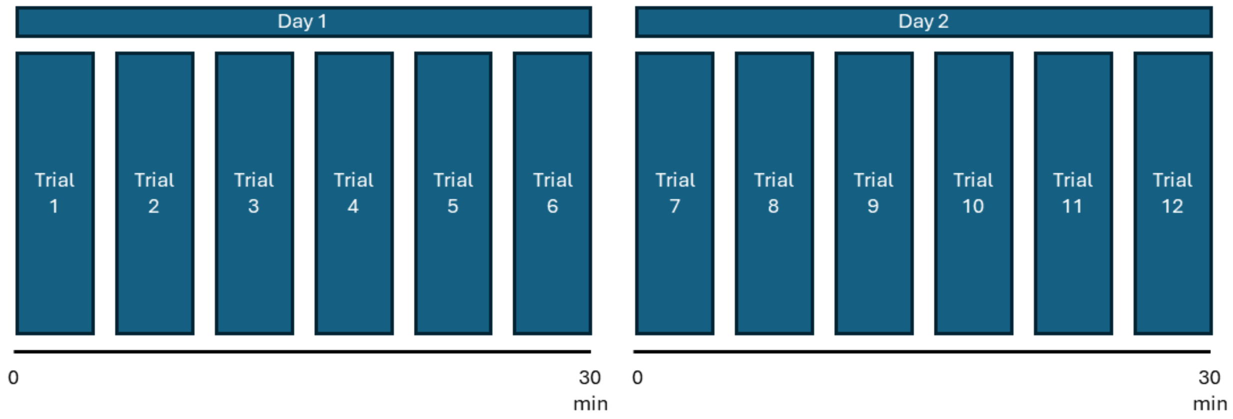

Figure. S2 A timeline of the test procedures. The trial order for each participant was different; it was randomized to mitigate order effects.

Postural response analysis

Since the Virtualis platform allows input signals only in the roll and pitch directions, the time-varying RALP and LARP displacement signals were spatially rotated 45 deg using a standard rotation matrix to provide the input signals via the Virtualis system.

$$\begin{bmatrix} SoS_{roll}\ (t) \\ SoS_{pitch}\ (t) \end{bmatrix} = \begin{bmatrix} \cos 45° & \sin 45° \\ -\sin 45° & \cos 45° \end{bmatrix} \begin{bmatrix} SoS_{LARP}\ (t) \\ SoS_{RALP}\ (t) \end{bmatrix}$$

Specifically, for Condition (ii), which delivered stimuli in the RALP and LARP directions, the roll signal resulting from the rotated and combined RALP and LARP signals was loaded as the roll input, and the pitch signal resulting from the rotated and combined RALP and LARP signals was loaded as the pitch input. For Condition (iii), the sum of the roll signal and the rotated roll signal from each of the RALP and LARP signals were loaded as the roll input. Similarly, the sum of the pitch signal and the rotated pitch signal from each of the RALP and LARP signals was loaded as the pitch input.

Results

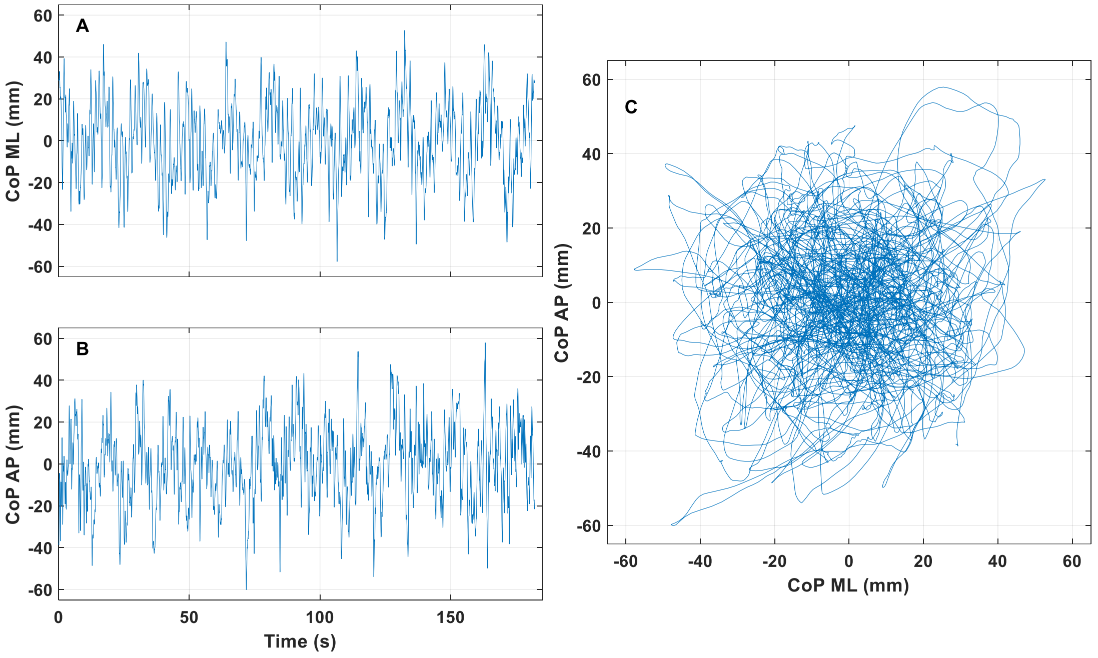

Figure. S3 Raw time traces of CoP ML (A) and AP (B) trajectories in one of the participants during Condition (i)-1 when 2D stimuli were provided in roll/pitch coordinates. Both CoP ML and AP trajectories are shown in the parametric plot (C).

Root Mean Square Displacement (RMSD) and Mean Velocity

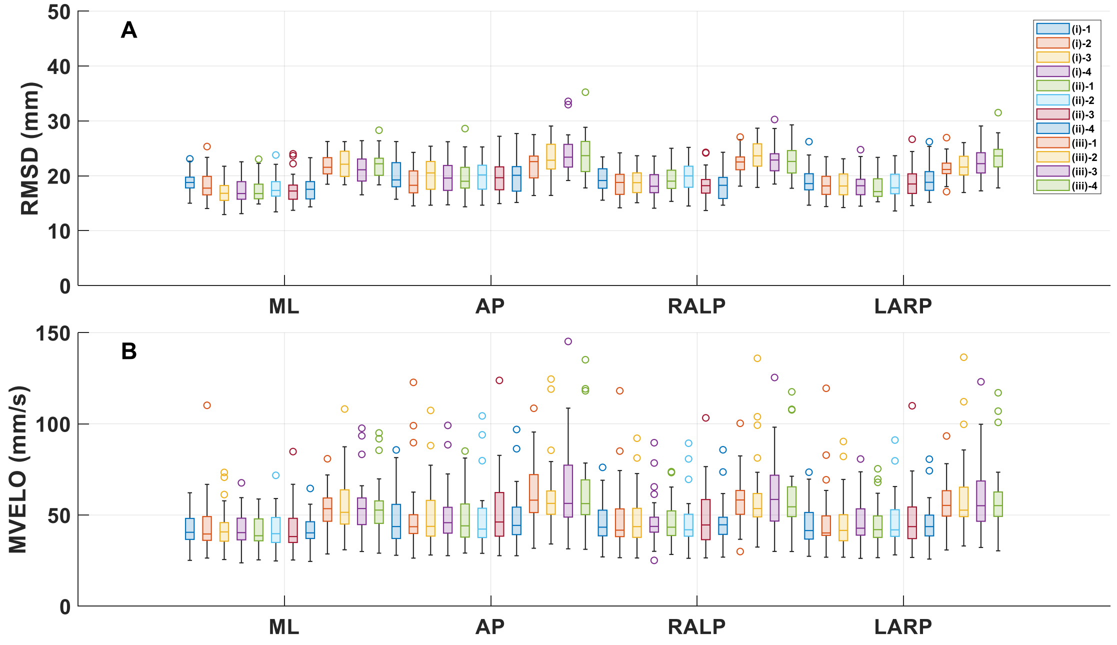

Figure. S4 The box-and whisker plots show the distribution of RMSD (A) and Mean Velocity (B) across the 24 subjects for each trial of each condition. Boxes show 25th to 75th percentile. The horizontal line that splits the box shows the median and the lines that extend from the box show the range of the data. The dots that appear past the ends of whiskers represent outliers. The 12 colors delineate the three condition that include the four trials that yield 12 trials in total.

Table. S1 Main effects and interaction effects of Condition and Trial on RMSD.

Dependent Variable	Comparison	F(df), p	Condition	AP Mean (SD)	ML Mean (SD)	RALP Mean (SD)	LARP Mean (SD)
RMSD (mm)	Condition	**F (8,88)= 11.84 p < 0.001**	(i)	19.663 (0.521)	17.795 (0.407)	18.972 (0.435)	18.592 (0.440)
			(ii)	19.857 (0.594)	17.711 (0.436)	19.000 (0.490)	18.649 (0.505)
			(iii)	23.312 (0.633)	21.827 (0.444)	22.938 (0.487)	22.290 (0.492)
	Trial	**F (12,204)= 6.69 p < 0.001**	Trial1	20.569 (0.569)	19.384 (0.410)	20.523 (0.445)	19.499 (0.467)
			Trial2	20.643 (0.554)	19.499 (0.496)	20.714 (0.517)	19.479 (0.461)
			Trial3	21.349 (0.634)	18.590 (0.460)	20.098 (0.506)	19.976 (0.551)
			Trial4	21.216 (0.681)	18.971 (0.469)	19.879 (0.537)	20.420 (0.558)
	Condition* Trial	**F (24,552) = 5.19 p < 0.001**	(i) Trial1	19.772 (0.613)	18.729 (0.432)	19.471 (0.489)	19.070 (0.526)
			(i) Trial2	18.782 (0.535)	18.391 (0.566)	18.825 (0.533)	18.398 (0.490)
			(i) Trial3	20.237 (0.618)	19.969 (0.447)	18.970 (0.529)	18.402 (0.507)
			(i) Trial4	19.863 (0.657)	17.093 (0.483)	18.621 (0.520)	18.499 (0.545)
			(ii) Trial1	19.8640. (0.678)	17.539 (0.467)	19.537 (0.534)	17.984 (0.515)
			(ii) Trial2	20.073 (0.591)	17.839 (0.502)	19.734 (0.564)	18.242 (0.486)
			(ii) Trial3	19.771 (0.636)	17.699 (0.541)	18.483 (0.529)	19.06 (0.619)
			(ii) Trial4	19.721 (0.670)	17.765 (0.508)	18.248 (0.550)	19.309 (0.590)
			(iii) Trial1	22.072 (0.582)	21.883 (0.426)	22.56 (0.436)	21.445 (0.457)
			(iii) Trial2	23.073 (0.634)	22.268 (0.520)	23.583 (0.533)	21.796 (0.487)
			(iii) Trial3	24.039 (0.748)	21.102 (0.522)	22.841 (0.568)	22.465 (0.607)
			(iii) Trial4	24.064 (0.796)	22.055 (0.491)	22.767 (0.605)	23.453 (0.619)

Table. S2 Main effects and interaction effects of Condition and Trial on mean velocity.

Dependent Variable	Comparison	F(df), p	Condition	AP Mean (SD)	ML Mean (SD)	RALP Mean (SD)	LARP Mean (SD)
Mean Velocity (mm/s)	Condition	**F (8,88) = 28.562 p < 0.001**	(i)	50.326 (3.634)	42.986 (2.213)	47.544 (2.998)	46.623 (2.919)
			(ii)	50.100 (0.509)	42.399 (1.997)	47.393 (2.823)	46.701 (2.773)
			(iii)	64.693 (4.791)	54.871 (2.961)	60.894 (3.926)	60.253 (3.889)
	Trial	**F (12,204) = 1.880 p = 0.038**	Trial1	53.249 (3.273)	45.650 (2.013)	50.825 (2.680)	49.544 (2.616)
			Trial2	55.182 (4.704)	47.579 (2.961)	51.964 (3.871)	51.936 (3.928)
			Trial3	57.108 (4.495)	47.357 (2.808)	53.534 (3.685)	52.462 (3.660)
			Trial4	54.619 (3.931)	46.422 (2.319)	51.451 (3.285)	50.828 (3.054)
	Condition* Trial	F (24,552) = 0.885 p = 0.62	(i) Trial1	48.540 (3.226)	41.977 (1.976)	46.374 (2.617)	45.110 (2.634)
			(i) Trial2	50.703 (4.557)	44.460 (3.492)	48.328 (4.029)	47.522 (4.037)
			(i) Trial3	51.559 (3.994)	42.944 (2.448)	48.192 (3.323)	47.214 (3.201)
			(i) Trial4	50.504 (3.441)	42.564 (2.026)	47.281 (2.939)	46.648 (2.599)
			(ii) Trial1	49.060 (3.114)	41.668 (1.920)	46.961 (2.510)	45.531 (2.553)
			(ii) Trial2	49.461 (3.787)	42.320 (2.158)	46.563 (3.042)	46.694 (3.022)
			(ii) Trial3	52.635 (4.286)	43.808 (2.822)	49.412 (3.503)	48.793 (3.609)
			(ii) Trial4	49.242 (3.324)	41.801 (1.808)	46.634 (2.684)	45.787 (2.516)
			(iii) Trial1	62.148 (3.676)	53.305 (2.414)	59.139 (3.124)	57.992 (2.889)
			(iii) Trial2	65.382 (5.921)	55.956 (3.594)	61.002 (4.736)	61.591 (4.939)
			(iii) Trial3	67.131 (5.491)	55.321 (3.371)	62.998 (4.495)	61.380 (4.414)
			(iii) Trial4	64.112 (5.276)	54.901 (3.367)	60.438 (4.479)	60.049 (4.277)

Table. S3 The peak-to-peak perturbation amplitudes of (i) roll/pitch, (ii) RALP/LARP, and (iii) roll/pitch/RALP/LARP stimuli

**Peak-to-peak (deg)**	**(i) roll/pitch**				**(ii) RALP/LARP**				**(iii) roll/pitch/RALP/LARP**			
	(i)-1	(i)-2	(i)-3	(i)-4	(ii)-1	(ii)-2	(ii)-3	(ii)-4	(iii)-1	(iii)-2	(iii)-3	(iii)-4
roll	1.11	1.00	0.89	0.96	1.14	1.35	1.29	1.25	2.04	1.96	1.53	2.00
pitch	0.96	0.89	1.11	1.00	1.21	1.22	1.24	1.40	1.93	1.70	2.09	1.81
RALP	1.35	1.14	1.25	1.29	1.00	1.11	0.96	0.89	1.96	2.04	2.00	1.53
LARP	1.22	1.21	1.40	1.24	0.89	0.96	1.00	1.11	1.70	1.93	1.81	2.09

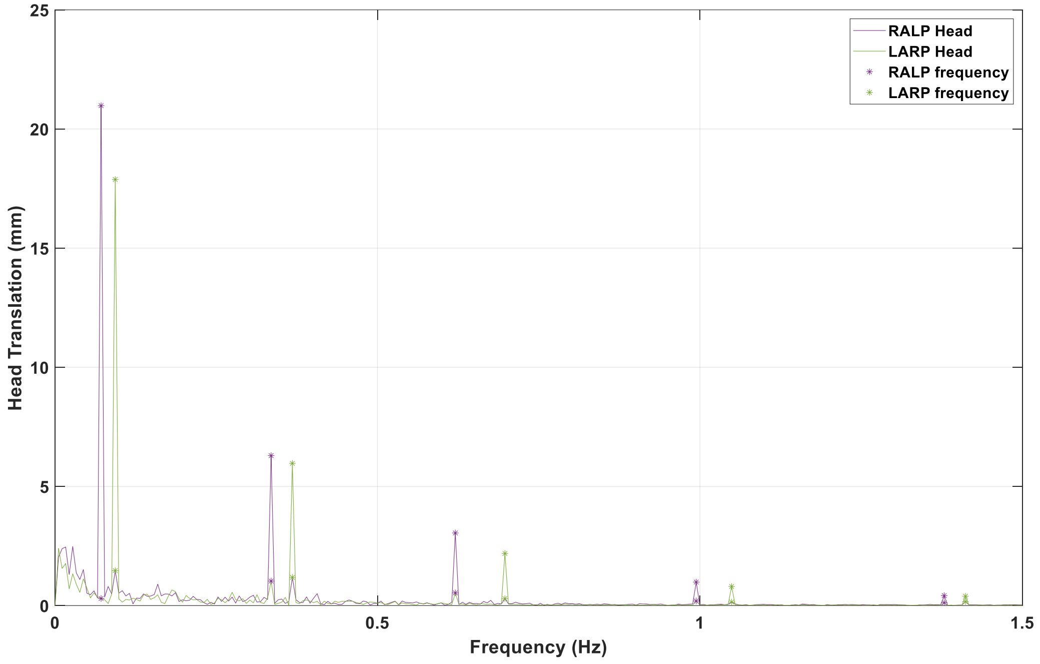

Figure. S5 Each of spectral head RALP and LARP linear average response components across the 24 subjects are identified at RALP frequencies and LARP frequencies for RALP/LARP Condition (ii)-1.

Table. S4 P-values of Hotelling T-squared tests on spectral responses at perturbed frequencies

Condition	Response	Stimuli	f_1	f_2	f_3	f_4	f_5
(i)-1	ML	roll	<0.0001	<0.0001	<0.0001	<0.0001	<0.0001
		pitch	0.6475	0.1423	0.0927	0.0085	0.5042
	AP	roll	0.5333	0.2124	0.3227	0.1457	0.6636
		pitch	<0.0001	<0.0001	<0.0001	<0.0001	<0.0001
	RALP	roll	<0.0001	<0.0001	<0.0001	<0.0001	<0.0001
		pitch	<0.0001	<0.0001	<0.0001	<0.0001	<0.0001
	LARP	roll	<0.0001	<0.0001	<0.0001	<0.0001	<0.0001
		pitch	<0.0001	<0.0001	<0.0001	<0.0001	<0.0001
(ii)-1	ML	RALP	<0.0001	<0.0001	<0.0001	<0.0001	<0.0001
		LARP	<0.0001	<0.0001	<0.0001	<0.0001	<0.0001
	AP	RALP	<0.0001	<0.0001	<0.0001	<0.0001	<0.0001
		LARP	<0.0001	<0.0001	<0.0001	<0.0001	<0.0001
	RALP	RALP	<0.0001	<0.0001	<0.0001	<0.0001	<0.0001
		LARP	0.1326	0.0002	0.0048	0.0128	<0.0001
	LARP	RALP	0.2121	0.0299	<0.0001	0.0218	0.0279
		LARP	<0.0001	<0.0001	<0.0001	<0.0001	<0.0001
(iii)-1	ML	roll	<0.0001	<0.0001	<0.0001	<0.0001	<0.0001
		pitch	0.0299	0.0729	0.2679	0.0003	0.0321
		RALP	<0.0001	<0.0001	<0.0001	<0.0001	<0.0001
		LARP	<0.0001	<0.0001	<0.0001	<0.0001	<0.0001
	AP	roll	0.0258	0.0523	0.1470	0.0382	0.3182
		pitch	<0.0001	<0.0001	<0.0001	<0.0001	<0.0001
		RALP	<0.0001	<0.0001	<0.0001	<0.0001	<0.0001
		LARP	<0.0001	<0.0001	<0.0001	<0.0001	<0.0001
	RALP	roll	<0.0001	<0.0001	<0.0001	<0.0001	<0.0001
		pitch	<0.0001	<0.0001	<0.0001	<0.0001	<0.0001
		RALP	<0.0001	<0.0001	<0.0001	<0.0001	<0.0001
		LARP	0.4029	0.0029	0.0827	<0.0001	0.0087
	LARP	roll	<0.0001	<0.0001	<0.0001	<0.0001	<0.0001
		pitch	<0.0001	<0.0001	<0.0001	<0.0001	<0.0001
		RALP	0.0628	0.1384	0.0017	0.7101	0.0121
		LARP	<0.0001	<0.0001	<0.0001	<0.0001	<0.0001

Sensitivity and Phase

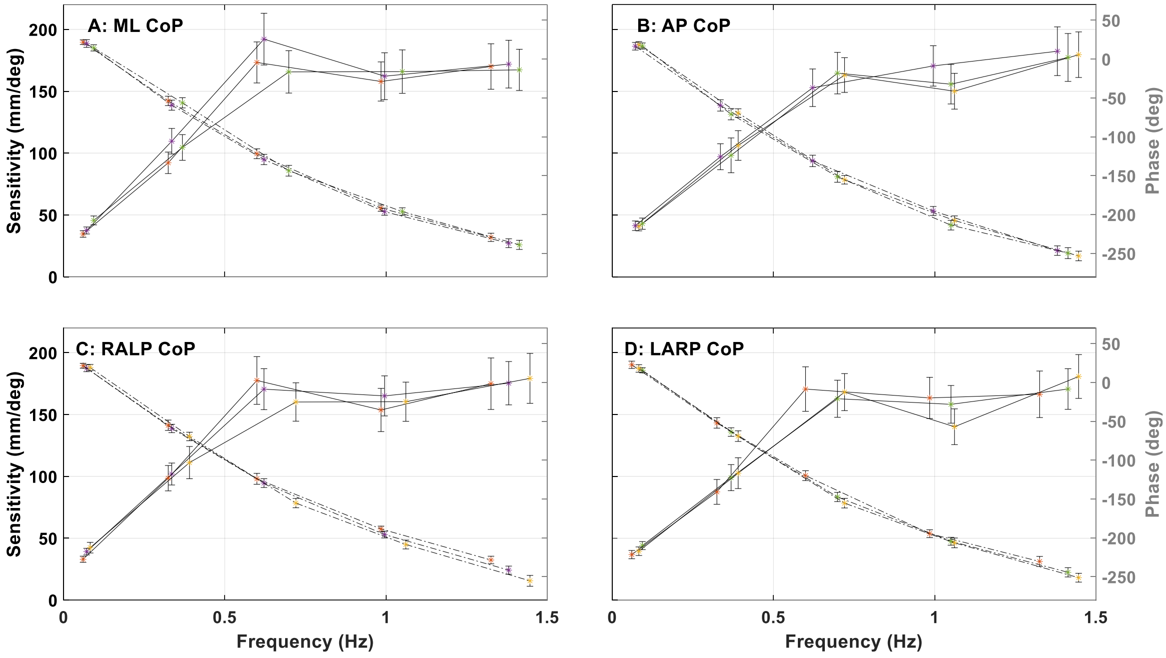

Figure. S6 The plots show the sensitivity (left y-axis) and phase (right y-axis) of the CoP response in the ML (A), AP (B), RALP (C), and LARP (D) planes versus perturbation stimuli frequency for Condition (iii) when SoS_{11} was provided for roll and SoS_{15} was provided for pitch, SoS_{13} was provided for RALP, and SoS_{17} was provided for LARP. Error bars show 95% confidence intervals. Different asterisk colors delineate the average sensitivity of CoP sway in response to roll (orange*), pitch (yellow*), RALP (purple*), and LARP (green*) stimuli.

Repeated Measures MANOVA

Table. S5 Main effects of the simultaneous stimuli, perturbation frequency, SoS trajectory, and dimension of the stimuli on the frequency response functions of CoP ML, AP, RALP, and LARP.

	**Simultaneous vs Separate (e.g., Condition (iii) vs (i))**	**Frequency (e.g., f1 vs f2)**	**SoS (e.g., SoS11 vs SoS13)**	**Dimension (i.e., roll/pitch vs RALP/LARP)**
	p	p	p	p
**ML**	< 0.001	< 0.001	< 0.001	0.63
**AP**	< 0.001	< 0.001	< 0.001	0.48
**RALP**	< 0.001	< 0.001	< 0.001	<0.001
**LARP**	< 0.001	< 0.001	< 0.001	<0.001

Roll/Pitch stimuli and RALP/LARP stimuli evoke similar AP, ML, RALP, and LARP responses.

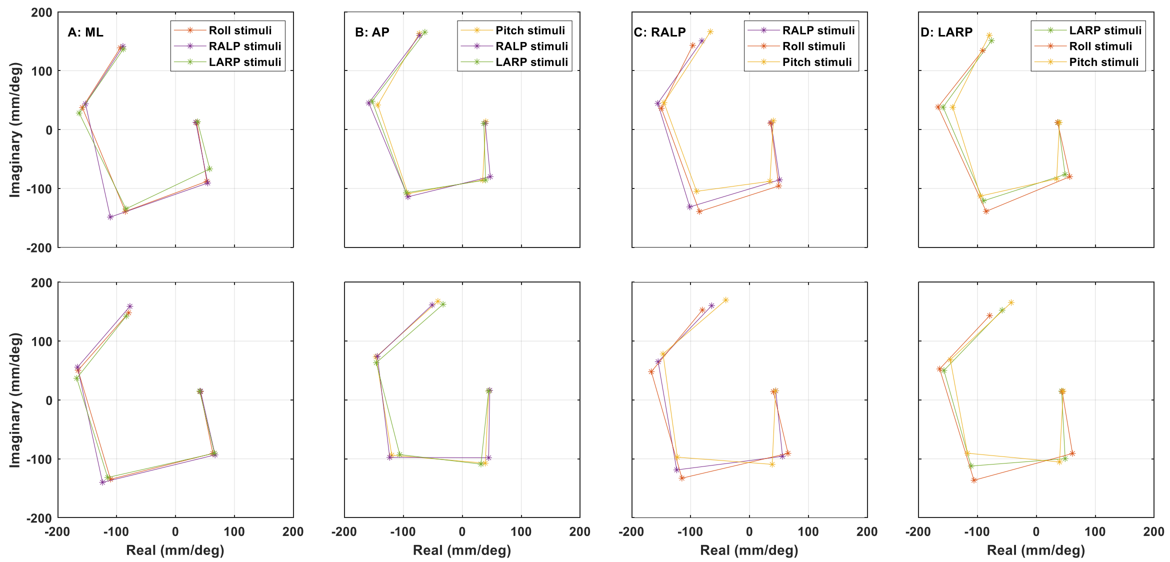

Figure. S7 The average frequency response functions of CoP (A) ML, (B) AP, (C) RALP, and (D) LARP in the complex plane across the 24 subjects (top) when simultaneous roll/pitch/RALP/LARP stimuli were provided using SoS_{13} and (bottom) when roll/pitch stimuli were provided using SoS_{13} on separate trials than RALP/LARP stimuli. Different asterisk colors delineate the average frequency responses to roll (orange*), pitch (yellow*), RALP (purple*), and LARP (green*) stimuli.

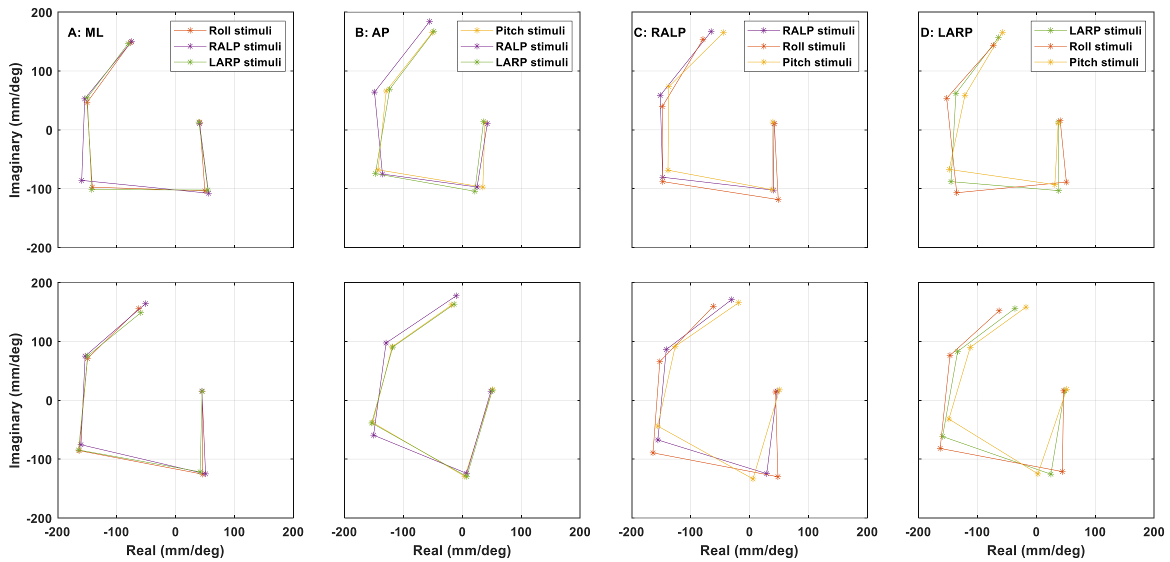

Figure. S8 The average frequency response functions of CoP (A) ML, (B) AP, (C) RALP, and (D) LARP in the complex plane across the 24 subjects (top) when simultaneous roll/pitch/RALP/LARP stimuli were provided using SoS_{15} and (bottom) when roll/pitch stimuli were provided using SoS_{15} on separate trials than RALP/LARP stimuli. Different asterisk colors delineate the average frequency responses to roll (orange*), pitch (yellow*), RALP (purple*), and LARP (green*) stimuli.

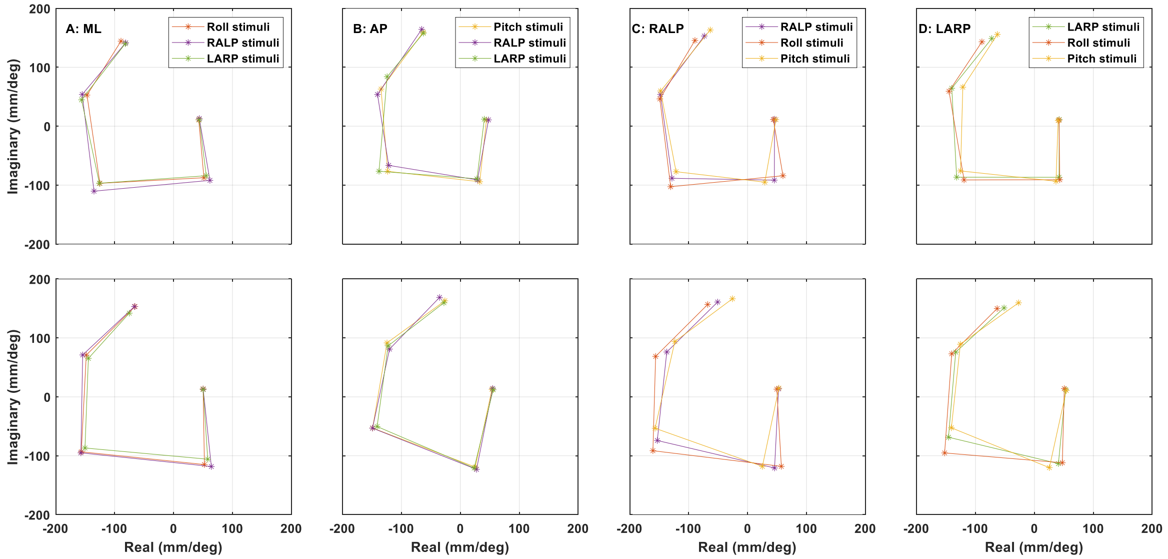

Figure. S9 The average frequency response functions of CoP (A) ML, (B) AP, (C) RALP, and (D) LARP in the complex plane across the 24 subjects (top) when simultaneous roll/pitch/RALP/LARP stimuli were provided using SoS_{17} and (bottom) when roll/pitch stimuli were provided using SoS_{17} on separate trials than RALP/LARP stimuli. Different asterisk colors delineate the average frequency responses to roll (orange*), pitch (yellow*), RALP (purple*), and LARP (green*) stimuli.

Discussion

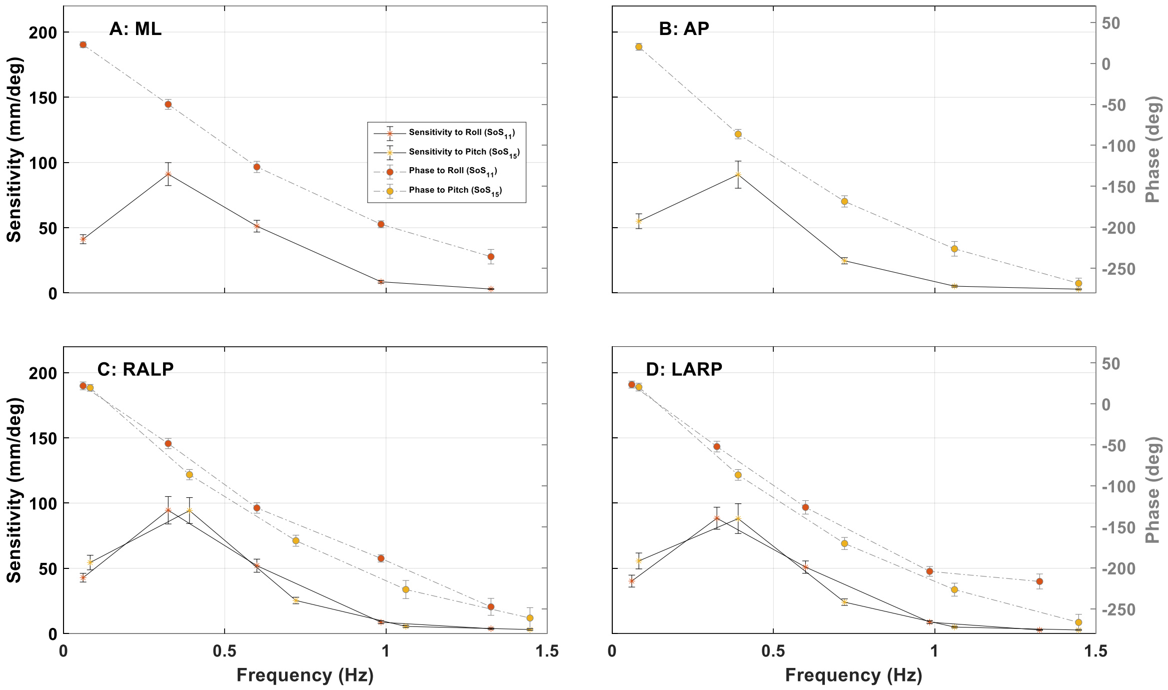

Figure. S10 The plots show the sensitivity (left y-axis) and phase (right y-axis) of the CoP response lowpass filtered with a cut-off frequency of 0.47 Hz (Peterka et al., 2018) to compare with earlier reports of CoM FRFs (Peterka 2002; Cenciarini and Peterka, 2006) in the ML (A), AP (B), RALP (C), and LARP (D) planes versus perturbation stimulus frequency for Test Condition (i) with SoS11 provided for roll and SoS15 provided for pitch. Error bars show 95% confidence intervals. Different asterisk colors delineate the average sensitivity of CoP sway in response to roll (orange*) and pitch (yellow*) stimuli.

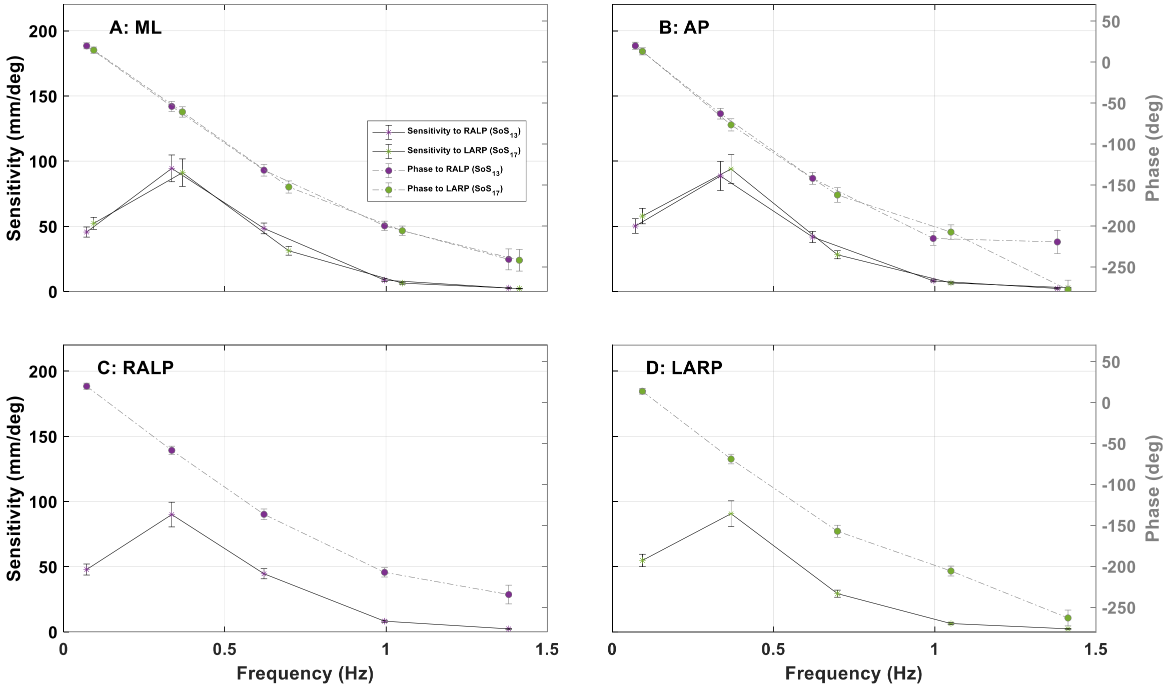

Figure. S11 The plots show the sensitivity (left y-axis) and phase (right y-axis) of the CoP response lowpass filtered with a cut-off frequency of 0.47 Hz (Peterka et al., 2018) to compare with earlier reports of CoM FRFs (Peterka 2002; Cenciarini and Peterka, 2006) in the ML (A), AP (B), RALP (C), and LARP (D) planes versus perturbation stimuli frequency for Test Condition (ii) with SoS13 provided for RALP and SoS17 provided for LARP. Error bars show 95% confidence intervals. Different asterisk colors delineate the average sensitivity of CoP sway in response to RALP (purple*) and LARP (green*) stimuli.

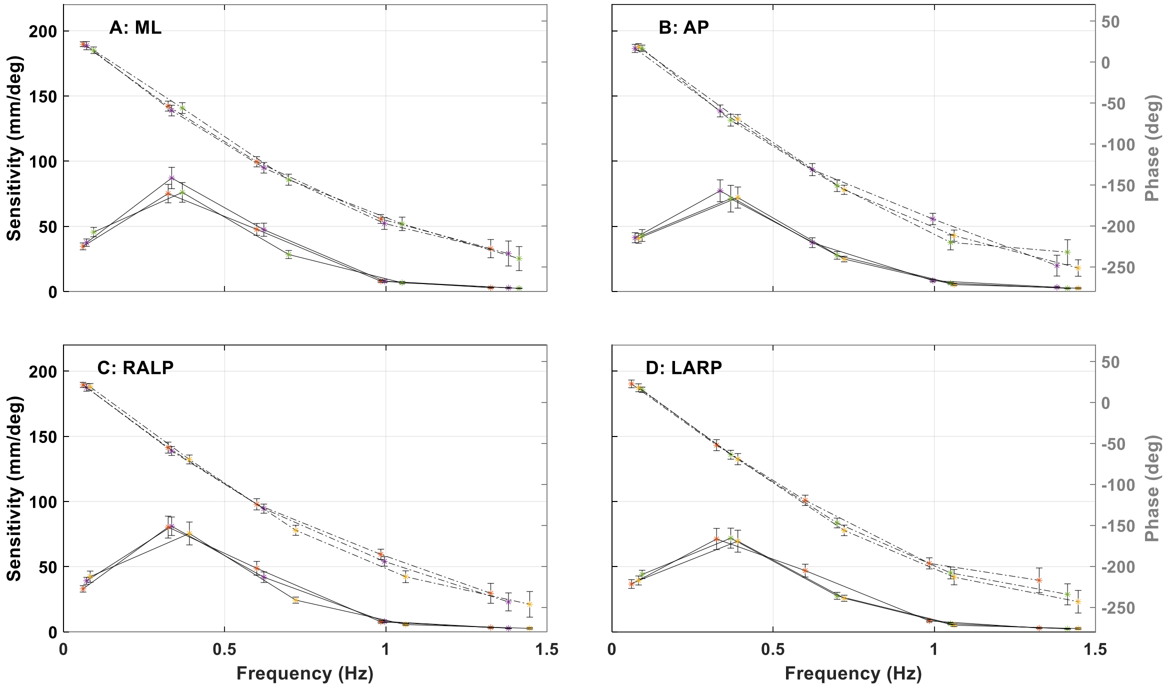

Figure. S12 The plots show the sensitivity (left y-axis) and phase (right y-axis) of the CoP response lowpass filtered with a cut-off frequency of 0.47 Hz (Peterka et al., 2018) to compare with earlier reports of CoM FRFs (Peterka 2002; Cenciarini and Peterka, 2006) in the ML (A), AP (B), RALP (C), and LARP (D) planes versus perturbation stimuli frequency for Condition (iii) when SoS_{11} was provided for roll and SoS_{15} was provided for pitch, SoS_{13} was provided for RALP, and SoS_{17} was provided for LARP. Error bars show 95% confidence intervals. Different asterisk colors delineate the average sensitivity of CoP sway in response to roll (orange*), pitch (yellow*), RALP (purple*), and LARP (green*) stimuli.
